# Genetic Predisposition to Chronic Lymphocytic Leukemia Is Mediated by a *BMF* Super-Enhancer Polymorphism

**DOI:** 10.1016/j.celrep.2016.07.053

**Published:** 2016-08-11

**Authors:** Radhika Kandaswamy, Georgina P. Sava, Helen E. Speedy, Sílvia Beà, José I. Martín-Subero, James B. Studd, Gabriele Migliorini, Philip J. Law, Xose S. Puente, David Martín-García, Itziar Salaverria, Jesús Gutiérrez-Abril, Carlos López-Otín, Daniel Catovsky, James M. Allan, Elías Campo, Richard S. Houlston

**Affiliations:** 1 Division of Genetics and Epidemiology, The Institute of Cancer Research, London SW7 3RP, UK; 2 Institut d’Investigacions Biomèdiques August Pi i Sunyer (IDIBAPS), 08036 Barcelona, Spain; 3 Departament d’Anatomía Patològica, Microbiología i Farmacología, Universitat de Barcelona, 08036 Barcelona, Spain; 4 Departamento de Bioquímica y Biología Molecular, Instituto Universitario de Oncología (IUOPA), Universidad de Oviedo, 33006 Oviedo, Spain; 5 Division of Molecular Pathology, The Institute of Cancer Research, London SW7 3RP, UK; 6 Newcastle Cancer Centre, Northern Institute for Cancer Research, Medical School, Newcastle University, Newcastle-upon-Tyne NE2 4HH, UK; 7 Unitat de Hematología, Hospital Clínic, IDIBAPS, Universitat de Barcelona, 08036 Barcelona, Spain

## Abstract

Chronic lymphocytic leukemia (CLL) is an adult B cell malignancy. Genome-wide association studies show that variation at 15q15.1 influences CLL risk. We deciphered the causal variant at 15q15.1 and the mechanism by which it influences tumorigenesis. We imputed all possible genotypes across the locus and then mapped highly associated SNPs to areas of chromatin accessibility, evolutionary conservation, and transcription factor binding. SNP rs539846 C>A, the most highly associated variant (p = 1.42 × 10^−13^, odds ratio = 1.35), localizes to a super-enhancer defined by extensive histone H3 lysine 27 acetylation in intron 3 of B cell lymphoma 2 (BCL2)-modifying factor (*BMF*). The rs539846-A risk allele alters a conserved RELA-binding motif, disrupts RELA binding, and is associated with decreased *BMF* expression in CLL. These findings are consistent with rs539846 influencing CLL susceptibility through differential RELA binding, with direct modulation of *BMF* expression impacting on anti-apoptotic BCL2, a hallmark of oncogenic dependency in CLL.

## Highlights

• SNP rs539846 underlies 15q15.1 association with chronic lymphocytic leukemia
• rs539846 resides in a B cell super-enhancer, disrupting a conserved RELA-binding site
• The rs539846 risk allele (A) reduces enhancer activity and RELA binding in CLL
• rs539846-A confers lower *BMF* expression

## Graphical Abstract

**Figure:**
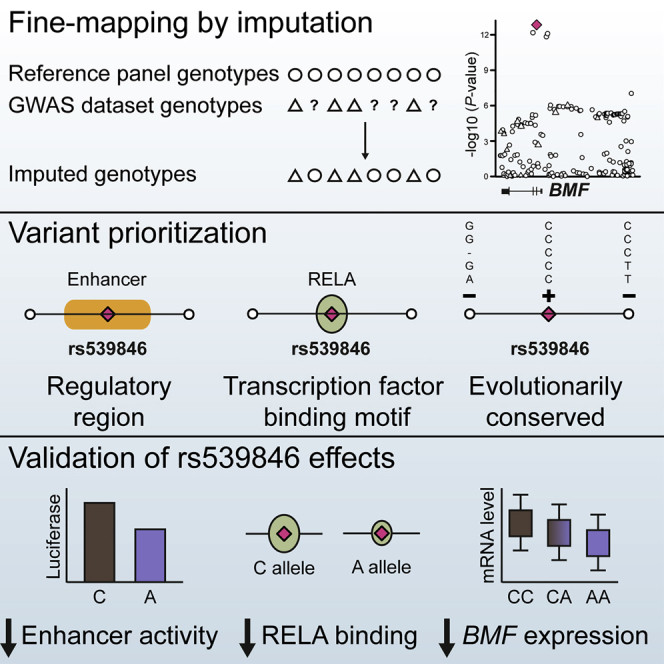

## Teaser

Kandaswamy et al. find that SNP rs539846 underlies the 15q15.1 chronic lymphocytic leukemia risk locus. Follow-up data demonstrate that rs539846 resides within a transcriptional enhancer and alters RELA binding at a conserved site. The rs539846-A risk allele results in reduced RELA-mediated enhancer activity and lower expression of BCL-2-modifying factor.

## Introduction

Although genome-wide association studies (GWASs) frequently have identified statistically significant associations within non-coding regions of the genome, the underlying causal variant has been elucidated in only a few instances. GWASs of chronic lymphocytic leukemia (CLL) have identified 31 risk loci, with the signal annotating B cell lymphoma 2 (BCL2)-modifying factor (*BMF*) at 15q15.1 being highly robust (Berndt et al., 2013, Berndt et al., 2016, Crowther-Swanepoel et al., 2010, Di Bernardo et al., 2008, Slager et al., 2011, Slager et al., 2012, Speedy et al., 2014).

Elevated expression of the anti-apoptotic protein BCL2 is a hallmark of CLL, driving the accumulation of mature leukemic lymphocytes (Hanada et al., 1993). BMF, a BH3-only pro-apoptotic member of the BCL2 protein family, neutralizes the anti-apoptotic activity of BCL2 through direct interaction (Puthalakath et al., 2001). Here we sought to identify the causal polymorphism(s) driving the 15q15.1 association with CLL susceptibility as a basis for understanding BCL2 addiction mechanisms in CLL.

## Results

### Fine-Mapping of the 15q15.1 CLL Risk Locus

**Figure 1:**
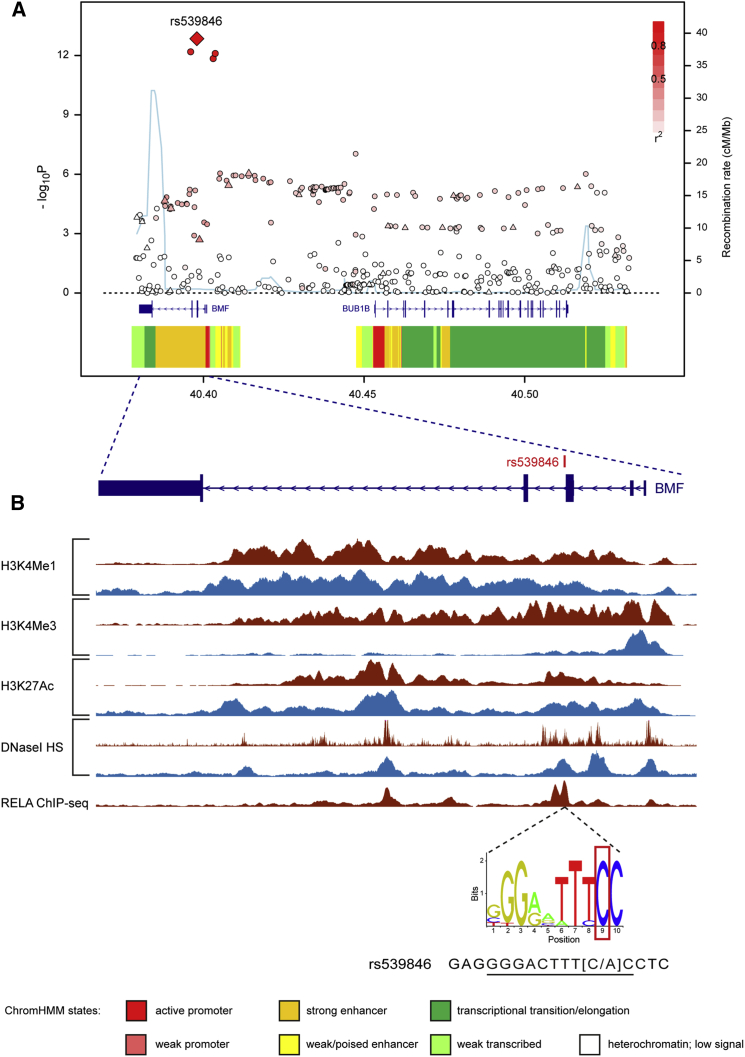
Genetic Mapping and Epigenetic Landscape at the 15q15.1 Locus (A) SNAP plot of the 15q15.1 chronic lymphocytic leukemia susceptibility locus. Genotyped (triangles) and imputed (dots) SNPs are shown based on their megabase chromosomal position on the x axis and -log10 p value on the y axis. Color intensity of each symbol reflects the extent of LD with rs539846 (white *r*^*2*^ = 0 to dark red *r*^*2*^ = to 1). Recombination rates, estimated using HapMap samples of European ancestry, are shown by a light blue line. Physical positions are based on NCBI build 37 of the human genome. Also shown are relative gene positions and chromatin state segmentation (ChromHMM) for GM12878 derived from ENCODE project data. (B) ChIP-seq data for H3K4Me1, H3K4Me3, and H3K27Ac histone modifications and DNaseI hypersensitivity (HS) are illustrated for GM12878 from ENCODE (brown) and for CLL cells from the Blueprint Project (blue). ChIP-seq data for RELA in GM12878 also is shown. Data are shown relative to the genomic arrangement of *BMF* and were plotted in the University of California, Santa Cruz Genome Browser. Also illustrated are the position-weighted matrix for RELA and the motif sequence (underlined) altered by rs539846 (red box).

A previous GWAS reported an association between rs8024033 at 15q15.1 and CLL risk (Berndt et al., 2013). To refine the association signal, we performed fine-mapping of the 15q15.1 CLL risk locus by imputation of our European GWAS to 1000 Genomes Project (Abecasis et al., 2012) and UK10K (UK10K Consortium et al., 2015) reference panels. By this approach, we identified four risk SNPs with minor allele frequency >0.01 and association p < 5.0 × 10^−7^ (Figure 1A; Table S1). The lead SNP, rs539846 (odds ratio = 1.35, p = 1.42 × 10^−13^), mapped to the third intron of *BMF* and was in high linkage disequilibrium (LD, r^2^ = 0.91) with the published SNP, rs8024033. We verified the fidelity of imputed rs539846 genotypes by Sanger sequencing in a subset of 176 CLL cases, demonstrating >95% concordance.

To rule out the existence of multiple statistical signals at the *BMF* locus, we repeated association testing conditional on rs539846 genotypes, observing no significant variants (most significant variant: rs181168015, p = 1.52 × 10^−4^; Figure S1). We also found no rare non-synonymous variants in *BMF* in the germline exomes of 141 CLL cases (enriched for genetic susceptibility by virtue of family history; Supplemental Experimental Procedures). Collectively these results are consistent with a single underlying variant at the 15q15.1 locus.

### Definition of rs539846 as a Plausible CLL Risk SNP

**Figure 2:**
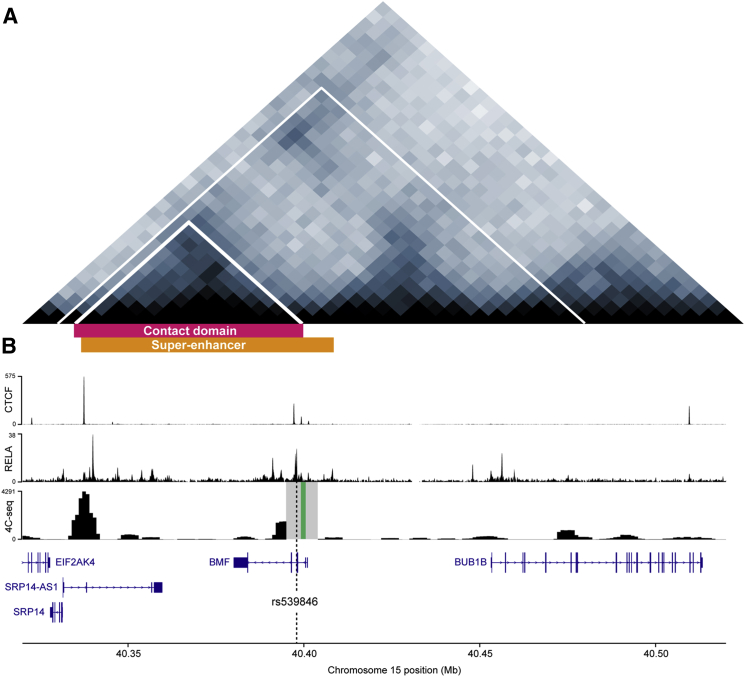
Contact Profile of the *BMF* 4C Viewpoint in Combination with ChIP-Seq Data (A) Heatmap representing chromatin interactions in GM12878 cells at 40.32–40.52 Mb on chromosome 15. Chromatin contact domains called by the Arrowhead algorithm are marked by white lines (Rao et al., 2014). The contact domain and CD19^+^ B cell super-enhancer (Hnisz et al., 2013) encompassing rs539846 are labeled in pink and yellow, respectively. (B) 4C-seq analyses in MEC1 CLL cells indicate the formation of a loop domain between rs539846 and *cis*-regulatory elements. The 4C viewpoint (green box) lies adjacent to rs539846 (dotted line). A 10-kb masked region (gray box) is also marked. ENCODE ChIP-seq data from GM12878 cells show the correspondence between loop formation and CTCF and RELA transcription factor occupancy. Canonical transcripts and chromosome 15 position also are shown.

To further prioritize candidate CLL risk variants, we examined the regulatory potential of SNPs in LD (r^2^ > 0.2) with rs539846, based on epigenetic data from lymphoblastoid cell lines (LCLs [ENCODE Project Consortium, 2012]) and primary CLL cells. These data showed that rs539846 resides within an active enhancer region inferred by DNaseI sensitivity and H3K4me1 and H3K27ac histone modifications in both cell types (Figure 1B; Table S1). Moreover, analysis of histone H3K27ac data from lymphoid cells of both B cell and T cell lineages defined a B cell-specific 15q15.1 super-enhancer that spans ∼80 kb, encompassing the CLL risk locus (Hnisz et al., 2013), while high-throughput chromosome conformation capture (Hi-C) data from LCLs (Rao et al., 2014) show that this putative super-enhancer element overlaps a chromatin contact domain (Figure 2A).

Since causal SNPs that drive GWAS associations may function by altering transcription factor binding, we examined whether 15q15.1 candidate risk SNPs disrupt predicted JASPAR motifs. This revealed that rs539846 alters a highly conserved base within a putative RELA-binding motif (GGGACTTT[C/A]C, phastCons score = 1.00, Genomic Evolutionary Rate Profiling [GERP] score = 4.81) (Figure 1B). Encyclopedia of DNA Elements (ENCODE) transcription factor chromatin immunoprecipitation sequencing (ChIP-seq) in LCLs confirmed the presence of RELA binding across this site (Figure 1B) in cells homozygous for the rs539846-C allele (non-risk allele, preserving the RELA motif). Within the 15q15.1 chromatin contact domain, chromosome conformation capture–on-chip with sequencing (4C-seq) in the MEC1 CLL cell line showed a high frequency of three-dimensional contacts between the viewpoint (adjacent to rs539846) and the distal end of the predicted super-enhancer, with both points overlapping regions of RELA and CTCF binding (Figure 2B).

No other candidate CLL risk variant at the 15q15.1 locus showed the unique combination of evolutionary conservation, active enhancer localization, and disruption of a transcription factor-binding motif, thus re-affirming that rs539846 is the single best causal SNP candidate.

### rs539846 Alters RELA-Mediated Enhancer Activity

**Figure 3:**
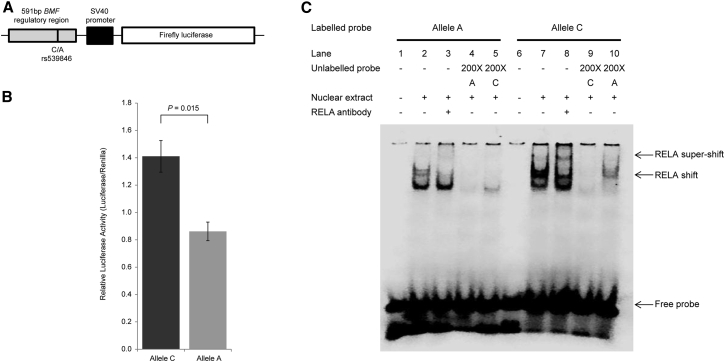
rs539846 Affects RELA-Bound Enhancer Activity (A) Allele-specific constructs containing a 591-bp putative regulatory sequence flanking rs539846 were cloned into the pGL3-promoter luciferase reporter vector. (B) The ratio of luminescence from the experimental pGL3-rs539846 constructs to the Renilla internal control, pRL-SV40, was normalized to the empty pGL3-SV40 vector. Data shown are mean ± SE from three independent experiments performed in triplicate. Difference in expression was assessed by the Student’s t test. The rs539846-A risk allele had significantly decreased enhancer activity over the protective allele. (C) EMSA showing differential binding of MEC1 nuclear protein to the rs539846-C allele (protective) and the rs539846-A allele (risk). Binding of double-stranded A allele and C allele probes to MEC1 nuclear extract shows a marked reduction of DNA-protein binding associated with the A allele.

We next performed luciferase reporter assays to determine the effect of rs539846 on enhancer activity. MEC1 cells transfected with constructs containing the risk A allele demonstrated a significant reduction in normalized luminescence compared to the C allele (p = 0.015, Figures 3A and 3B), indicating that the intact RELA motif is required for enhancer activity. We assayed protein-DNA interactions for rs539846-C and -A alleles using electrophoretic mobility shift assays (EMSAs). The C allele formed stronger protein-DNA complexes compared with the A allele (Figure 3C), and in an EMSA super-shift assay RELA was preferentially recruited to the C allele (Figure 3C).

### rs539846 Alters RELA-Mediated Regulation of *BMF*

**Figure 4:**
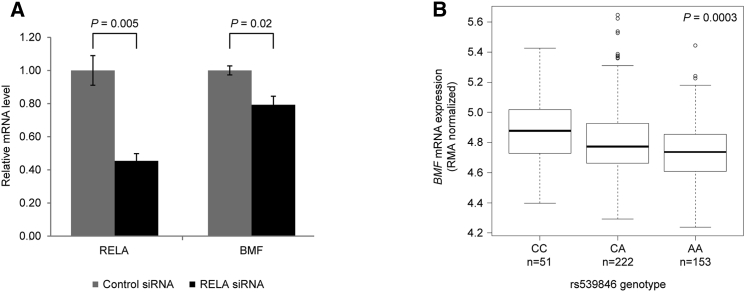
RELA Expression and the rs539846 Genotype Are Correlated with *BMF* Transcript Levels (A) siRNA knockdown of RELA reduces transcript levels of *BMF*. Data shown are mean ± SE for three independent replicates relative to GAPDH reference mRNA, normalized to control siRNA. The p values were determined with a two-tailed t test. (B) The rs539846 CLL risk allele (A) was associated with lower *BMF* mRNA levels in 462 patient samples. Boxplot indicates the median (horizontal line), first to third quartiles (box), and 1.5 times the interquartile range (whiskers) of *BMF* expression.

To determine whether *BMF* is a target of RELA-mediated regulation, we first queried the International Cancer Genome Consortium (ICGC) dataset, revealing a correlation between *RELA* and *BMF* expression in CLL (Table S2; p = 0.004). To establish a direct relationship between *RELA* and *BMF* expression, we performed small interfering RNA (siRNA) experiments in MEC1 cells, where knockdown of *RELA* was accompanied by a significant reduction in *BMF* mRNA (Figure 4A, p = 0.02; Figure S2). We also investigated whether the rs539846 genotype was associated with *BMF* transcript levels in 426 primary CLL cases. We observed a significant dose relationship between the rs539846-A risk allele and reduced *BMF* mRNA (p = 0.0003; Figure 4B). No association was seen between the rs539846 genotype and levels of other genes within 1 Mb of the SNP (false discovery rate < 0.05, Table S2). To investigate whether the rs539846 genotype might influence splicing of *BMF*, we examined RNA sequencing (RNA-seq) data from 30 CLL cases, finding no evidence of aberrant splicing. We also found no differences in the splicing levels of known *BMF* exons between the rs539846 risk allele and non-risk allele homozygotes (Figure S3).

### Impact of rs539846 on Prognosis and Survival in CLL Patients

CLL can be classified on the basis of several prognostic factors, including immunoglobulin heavy-chain variable (*IGHV*) mutation status; expression levels of CD38, ZAP70, and CLLU1; as well as somatic genomic abnormalities (trisomy 12, 13q14 deletion, 6q21 deletion, 11q23 deletion, 17p13 deletion, *NOTCH1* mutation, and *SF3B1* mutation). We found no association between the rs539846 genotype and these features in a subset of UK-GWAS and ICGC study cases (Table S3). There was also no association between rs539846 and overall patient outcome (Table S4), and we noted that *BMF* transcript levels were not associated with patient survival (Table S4).

## Discussion

Collectively, our data demonstrate that the underlying molecular mechanism for the 15q15.1 CLL risk locus is mediated through rs539846, which resides within a transcriptional enhancer and disrupts a conserved RELA transcription factor-binding site. Our data are compatible with the rs539846-A allele conferring increased CLL risk through reduced RELA-mediated expression of the pro-apoptotic BCL2 family gene, *BMF*. Furthermore, epigenetic and chromosome conformation capture data are consistent with rs539846 localizing within a chromatin contact domain, overlapping a B cell super-enhancer (Hnisz et al., 2013). This interval, anchored by divergent CTCF-binding sites, forms a loop domain (Rao et al., 2014), which is expected to bring two regions of RELA binding, separated by a linear distance of around 65 kb, into physical contact close to the promoter of *BMF*.

RELA (also known as p65) is a sub-unit of the necrosis factor kappa B (NF-κB) protein complex. This transcription factor complex regulates expression of genes involved in biological processes, such as proliferation, survival, and inflammation. NF-κB signaling is constitutively active in CLL (Furman et al., 2000), while high levels of the pro-survival gene *BCL2*, an established NF-κB target, are a hallmark of the disease (Scarfò and Ghia, 2013).

Here, we provide direct evidence that *BMF* is transcriptionally regulated by RELA, in keeping with the somewhat counter-intuitive observation that levels of pro-apoptotic BMF are high in CLL (Mackus et al., 2005). In the normal response to cellular stress, BMF interacts with BCL2 at the mitochondrial surface and neutralizes its anti-apoptotic properties (Puthalakath et al., 2001). In CLL, it is hypothesized that, although cells maintain some ability to induce pro-apoptotic BH3-only proteins like BMF in response to oncogenic stress, apoptosis ultimately fails due to overexpression of pro-survival proteins.

Our data suggest that, in individuals carrying the rs539846 risk allele, *BMF* transcript levels are reduced and thus the apoptotic response may be attenuated further. Indeed, previous studies in the myeloma cell line U266 have reported that siRNA-mediated knockdown of *BMF* is associated with a decrease in apoptosis following treatment with arsenic trioxide (Morales et al., 2008), whereas mice lacking *Bmf* develop a B cell lymphadenopathy caused by a resistance of B cells to apoptosis (Labi et al., 2008).

We did not observe an association between the SNP and prognostic markers or patient survival in CLL. This is consistent with differential expression of *BMF* being important in the early phases of CLL rather than disease progression per se. We do, however, acknowledge that our analysis had <50% power to demonstrate a 10% difference in patient outcome, and to robustly determine the relationship between *BMF* expression and patient outcome requires much larger patient cohorts.

Finally, this study underlines the importance of BH3-only proteins such as BMF in CLL development. Recently, a number of BH3 mimetics have been developed as potential therapies for lymphoid malignancies (Billard, 2013). These molecules are designed to mimic endogenous BH3-only proteins and bind to pro-survival members of the BCL2 family, facilitating the induction of apoptosis. One example, ABT-199 (Venetoclax), selectively targets BCL2, and, in recent clinical trials involving relapsed or refractory CLL, patients gave an overall response rate of 79% (Roberts et al., 2016). Our findings thus further demonstrate the utility of association studies to define clinically relevant oncogenic pathways.

## Experimental Procedures

### Ethics

Ethical approval for this study was obtained from the UK Multi-Research Ethics Committee (MREC 99/1/082).

### Fine-Mapping of the 15q15.1 Locus

We made use of data from two published CLL GWASs: (1) UK-CLL-1 (Di Bernardo et al., 2008), a scan of 517 cases using Illumina HumanCNV370-Duo BeadChips, with Hap1.2M-Duo Custom array data on 2,698 individuals from the Wellcome Trust Case Control Consortium 2 (WTCCC2) 1958 Birth cohort serving as controls; and (2) UK-CLL-2 (Speedy et al., 2014), a scan of 1,403 cases using the Illumina Omni Express BeadChips, with Hap1.2M-Duo Custom array data on 2,501 individuals from the UK Blood Service Control Group serving as controls. Individuals with low call rate (<90%), extremely high or low heterozygosity (p < 1.0 × 10^−4^), and those evaluated to be of non-European ancestry (using HapMap version 2 populations as a reference) were excluded.

GWAS data were imputed using 1000 Genomes Project phase 1 integrated release 3 (Abecasis et al., 2012), and UK10K 2014 release (UK10K Consortium et al., 2015) as a reference in conjunction with IMPUTE2 v2.1.1 software (Howie et al., 2009). Genotypes were aligned to the positive strand in both imputation and genotyping. Poorly imputed SNPs defined by an information measure, Is < 0.80, were excluded. The association between each SNP and CLL risk was assessed by Cochran-Armitage trend test. To look for independent effects, conditional logistic regression analysis was performed. SNP rs539846 was included as a covariate and association statistics for SNPs within the interval chr15:40379030–40532514 were recalculated (region spans all SNPs in LD r^2^ > 0.2 with rs539846). To validate imputed rs539846 genotypes, we performed Sanger sequencing in 176 CLL GWAS cases. Primers are listed in Table S5.

### Epigenetic Annotation

To explore the epigenetic profile of the interval, we examined LCL chromatin state segmentation, DNase sequencing (DNase-seq), histone modification, and transcription factor ChIP-seq data from the ENCODE project (ENCODE Project Consortium, 2012, Ernst and Kellis, 2012). In addition, ChIP-seq (H3K4me3, H3K4me1, and H3K27ac) and DNase-seq data generated using standard protocols within the Blueprint Consortium, from cells of a CLL patient with mutated *IGHV* (>90% tumor cell content), also were examined (Puente et al., 2015). Detailed protocols are available from the Blueprint Consortium (http://www.blueprint-epigenome.eu). We also used HaploReg (Ward and Kellis, 2012) to examine whether rs539846 or proxy SNPs (*r*^2^ > 0.2 in 1000 Genomes EUR reference panel) annotate transcription factor-binding sites or enhancer elements. We assessed sequence conservation using GERP (Cooper et al., 2005) and PhastCons (Siepel et al., 2005). We searched for overlap with annotated super-enhancer regions in lymphoid cell types from B cell (CD19^+^ and CD20^+^) and T cell (CD4^+^ naive and memory; CD8^+^ naive and memory) lineages (Hnisz et al., 2013).

### Hi-C and Definition of a Topological Domain at the 15q15.1 Locus

We made use of publicly available Hi-C data on GM12878 cells (Rao et al., 2014), based on combined replicates digested using MboI, analyzed using the balanced Knight-Ruiz normalization method (Knight and Ruiz, 2013) with a uniform resolution of 5 kb. Contact domains were defined with the Arrowhead algorithm (Rao et al., 2014).

### Cell Culture

MEC1 (human CLL) cells were grown in Iscove’s modified Dulbecco’s medium (Life Technologies) supplemented with 10% fetal calf serum.

### 4C-Seq

4C-seq libraries were prepared as described (van de Werken et al., 2012), using ten million MEC1 cells cross-linked with 2% formaldehyde. Using 4C primer design software (http://mnlab.uchicago.edu/4Cpd/), we identified a viewpoint adjacent to SNP rs539846. Primary and secondary restriction enzymes were *Dpn*II and *Hind*III (cut sites, chr15:40,397,659–40,397,662 bp and chr15:40,396,692–40,396,697 bp, respectively). Primers are listed in Table S5. Libraries were sequenced on an Illumina MiSeq to obtain 150-bp single-end reads. Reads were mapped to the human genome using Bowtie (version 2.1.0) and filtered for PHRED score < 30. Implementing standard procedures, unique 4C-seq reads were allocated to blind and non-blind fragments. Profiles for the two classes of fragments were obtained at 100-bp resolution and an average profile for a 5-kb running window was computed. For data visualization, we used Vispig (Scales et al., 2014) and incorporated processed ChIP-seq data from the ENCODE Project (ENCODE Project Consortium, 2012).

### Plasmid Construction and Luciferase Assays

Allele-specific fragments of a 591-bp region spanning rs539846 were amplified from human genomic DNA using primers detailed in Table S5, cloned into the PCR8/GW/TOPO vector, and then transferred into pGL3 *luc2* promoter vector using Gateway technology (Life Technologies). Reporter constructs were introduced into MEC1 cells by nucleofection, using program X-01 on the Amaxa Nucleofector I (Amaxa Biosystems). Typically, 5 × 10^6^ cells were resuspended in 100 μl Cell Line Nucleofector Solution V and mixed with 3 μg reporter plasmid DNA and 60 ng internal control plasmid (pRL-SV40). Transiently transfected cells were grown for 24 hr before assaying with the Dual-Luciferase Reporter Assay System (Promega) and the Fluoroskan Ascent FL plate reader (Labsystems). Relative luciferase activity was calculated as the ratio of luminescence from the experimental reporter to that of the control reporter. Each transfection experiment was repeated three times and statistical significance was calculated using the Student’s t test.

### EMSA

Nuclear protein was extracted from MEC1 cells using NE-PER nuclear and cytoplasmic extraction kits (Thermo Fisher Scientific). Infrared dye DY-682-labeled (Eurofins Genomics) and unlabeled (Life Technologies) complementary oligonucleotides flanking rs539846 (5′-GAGGGGACTTT[C/A]CCTCCCCAAAC-3′ and 5′-GTTTGGGGAGG[G/T]AAAGTCCCCTC-3′) were annealed to generate double-stranded EMSA probes. Each 20 μl binding reaction contained 50 fmol labeled target DNA, 1× binding buffer (10 mM Tris, 50 mM KCl, 1 mM DTT [pH 7.5], 1 μg poly [dI.dC, Sigma-Aldrich], 2.5 mM DTT, and 10 μg nuclear protein extract). The reaction mix was incubated in the dark for 30 min at room temperature. Competition assays were performed by adding 200-fold molar excess of unlabeled probes to the binding reaction. Super-shift EMSAs were conducted by adding 2 μg RELA antibody (Santa Cruz Biotechnology) to the binding reaction and incubating for 15 min prior to the addition of labeled probe. Post-incubation, 2 μl 10× Orange loading dye (LI-COR Biosciences) was added to the reaction mix, and the DNA-protein complexes were resolved by electrophoresis on a 6% DNA retardation gel (Life Technologies) in 0.5× Tris-borate-EDTA (TBE) at 4°C. Gels were imaged using the Odyssey Fc Infrared Imaging System (LI-COR Biosciences).

### Gene Expression and Splicing Analysis

We used Spearman’s rank correlation to assess the relationship between *BMF* and *RELA* transcript levels in the ICGC dataset (Puente et al., 2015). Expression quantitative trait locus analyses were performed for all genes in the 1-Mb region around rs539846, using Affymetrix Human Genome Array U219 data on 426 CLL patients (Puente et al., 2015). Four cases with 15q15.1 copy number losses were excluded. Genotypes were determined by imputation as described and were confirmed from whole-genome-sequencing (WGS) data in 145 samples, with >99% concordance. The association between SNP genotype and expression was evaluated by linear regression controlling for false discovery using matrixEQTL (Shabalin, 2012) implemented in R (version 3.2.0). To assess the impact of rs539846 on splicing, we used RNA-seq data from CLL tumors (Puente et al., 2015), counting individual k-mers supporting each of the possible splicing events. Sample genotype for SNP rs539846 was determined from WGS data, and differences between k-mer counts for rs539846-CC and -AA homozygotes were evaluated using a Student’s t test.

The accession numbers for the data utilized in this paper are European Genome-phenome Archive EGAD00010000875 and EGAS00000000092.

### siRNA Knockdown

siRNA targeting RELA and a control siRNA (Table S5) were obtained from Eurofins Genomics. MEC1 cells were transfected with 100 nM siRNA using nucleofection as described. Total RNA was extracted 24 hr post-transfection using the RNeasy Plus Mini Kit (QIAGEN). The cDNA was produced using SuperScript II Reverse Transcriptase (Life Technologies). Knockdown efficiency was measured by qPCR and western blot using standard protocols. RELA antibody was used with GAPDH antibody (FL-335; sc-25778, horseradish peroxidase [HRP]; Santa Cruz Biotechnology) as the loading control. Transcript levels of *RELA* and *BMF* were quantified using SYBR Green PCR mastermix (Life Technologies) and normalized to *GAPDH*. The experiment was repeated three times. Primer sequences are detailed in Table S5.

### Association between the rs539846 Genotype and Clinical Variables

Logistic regression was used to test the association between the rs539846 genotype and prognostic factors. Trisomy 12, 13q14 deletion, 6q21 deletion, 11q23 deletion, 17p13 deletion, CD38 expression, ZAP70 expression, CLLU1 expression, *NOTCH1* mutation, and *SF3B1* mutation statuses were determined in a subset of UK-CLL-1 patients who were participants in the LRF CLL4 Trial (Catovsky et al., 2007), as previously described (Gonzalez et al., 2013, Oscier et al., 2010, Oscier et al., 2013). *IGHV* mutation status was determined as per BIOMED-2 protocols (van Dongen et al., 2003) in a subset of patients from UK-CLL-1, UK-CLL-2, and the ICGC CLL project (Puente et al., 2015). In accordance with published criteria (van Krieken et al., 2007), we classified sequences with germline homology of ≥98% as unmutated and those with homology <98% as mutated. Survival analysis was performed using data from a subset of UK-CLL-1 and ICGC CLL project cases. Analysis was carried out using the log-rank test (using time from diagnosis to death or censoring at the end of follow-up).

## Author Contributions

R.S.H. designed the study and drafted the manuscript with contributions from all other authors. R.K., G.P.S., H.E.S., and J.B.S. performed the experiments. P.J.L., H.E.S., R.K., G.P.S., and G.M. performed the bioinformatics analysis. D.C. and J.M.A. performed sample recruitment. R.S.H. obtained financial support. In Spain, S.B., J.I.M.-S., D.M.-G., I.S., X.S.P., and J.G.-A. performed data analysis. C.L.-O. and E.C. supervised the data analysis.

## Supplemental Information

Document S1. Supplemental Experimental Procedures, Figures S1–S3, and Table S1–S5

Document S2. Article plus Supplemental Information
