# Supplementary material for: Genetic Predisposition to Chronic Lymphocytic Leukemia Is Mediated by a BMF Super-Enhancer Polymorphism
Source: Cell Rep. 2016 Aug 11;16(8):2061–7. doi: 10.1016/j.celrep.2016.07.053 (PMC4999417; doi:10.1016/j.celrep.2016.07.053)
Supplement: Document S1. Supplemental Experimental Procedures, Figures S1–S3, and Table S1–S5 [file mmc1.pdf]

Cell Reports, Volume *16*

Supplemental Information

Genetic Predisposition to Chronic Lymphocytic Leukemia Is Mediated by a *BMF* Super-Enhancer Polymorphism

Radhika Kandaswamy, Georgina P. Sava, Helen E. Speedy, Sílvia Beà, José I. Martín-Subero, James B. Studd, Gabriele Migliorini, Philip J. Law, Xose S. Puente, David Martín-García, Itziar Salaverria, Jesús Gutiérrez-Abril, Carlos López-Otín, Daniel Catovsky, James M. Allan, Elías Campo, and Richard S. Houlston

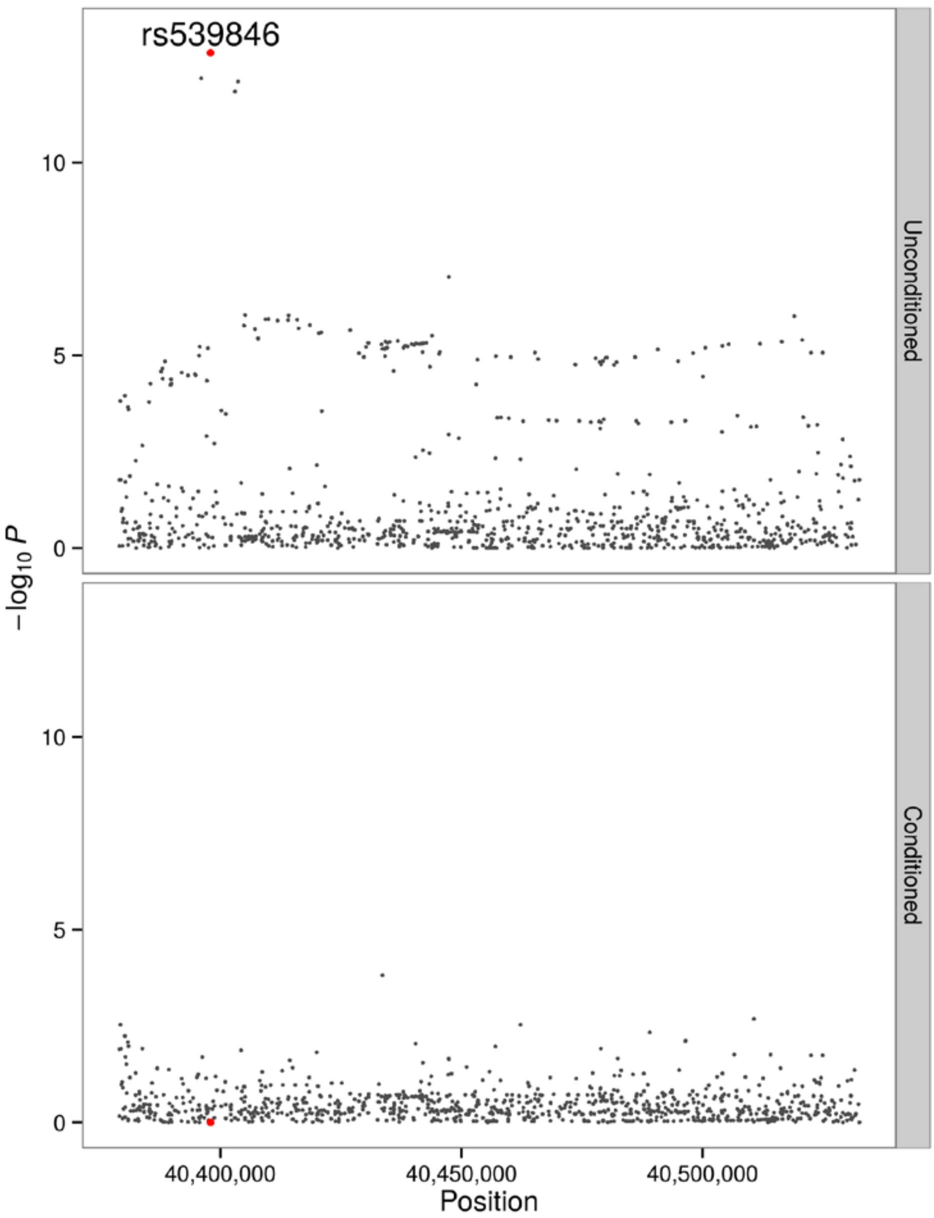

Figure S1, related to Figure 1. Conditional analysis plot for SNP rs539846.
Top panel shows results of unconditioned CLL association analysis for SNPs between 40.38 and 40.53Mb on chromosome 15. Bottom panel shows analysis conditioned on rs539846. SNP position is plotted on the x-axis and –log10 *P*-value is plotted on the y-axis.

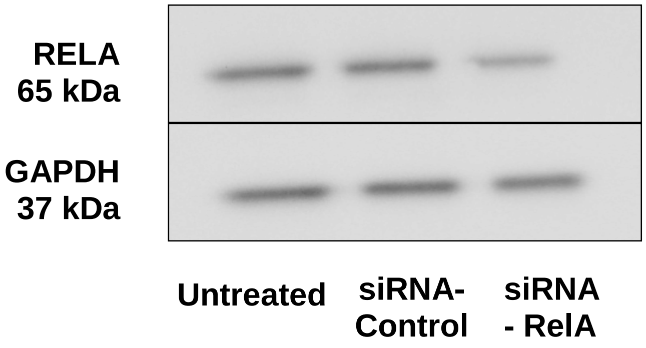

Figure S2, related to Figure 4. siRNA Knockdown of RELA.
Western blot analysis of RELA expression in MEC1 cells 24h post-transfection with indicated siRNAs.

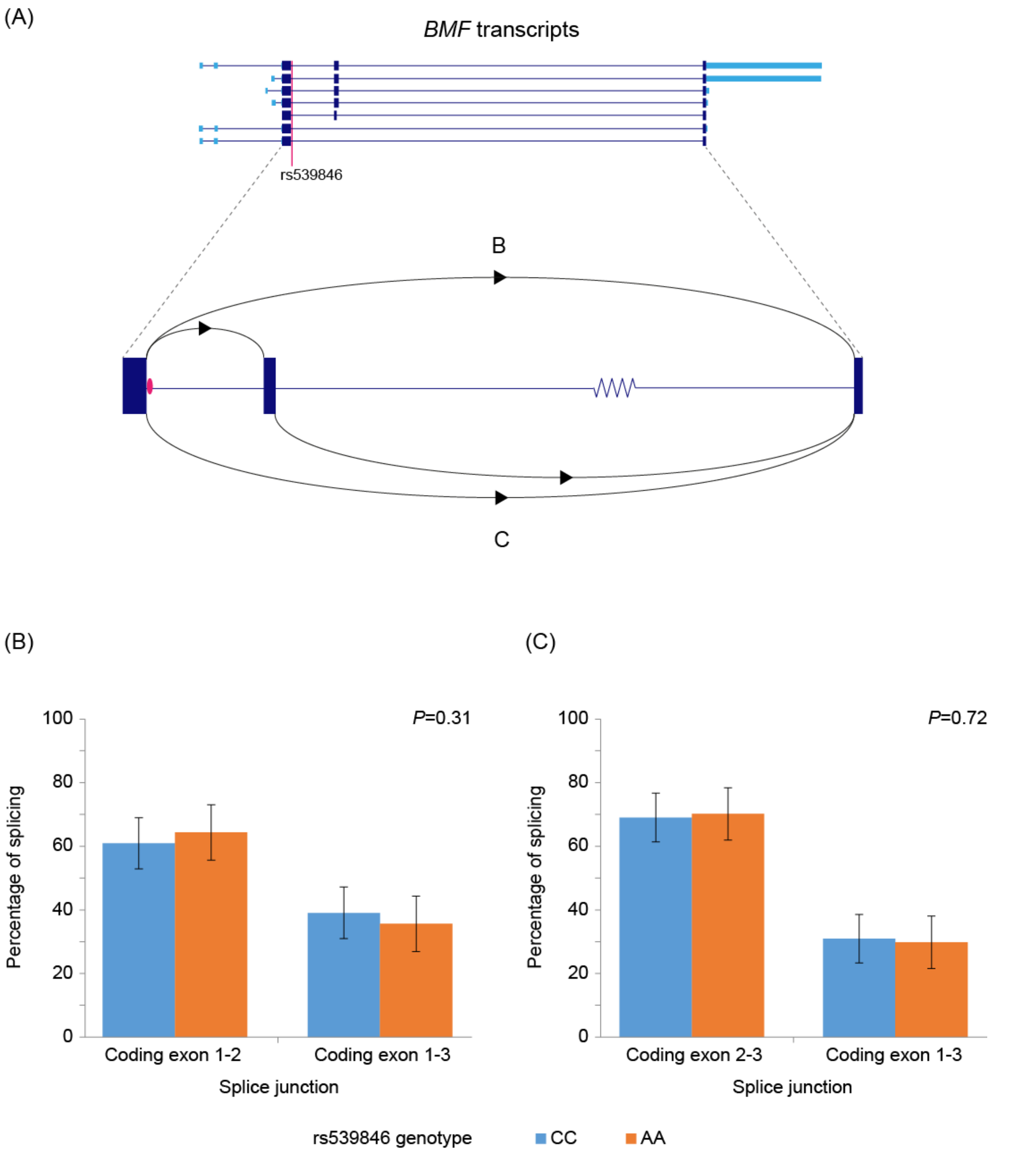

Figure S3, related to Figure 4. *BMF* splicing in CLL cases
(A) Multiple *BMF* transcripts encode three protein isoforms. The second coding exon encodes the BH3 protein domain and may be short-length, full-length or absent. The intronic SNP rs539846 (pink line/dot) resides between coding exon 1 and coding exon 2. Dark blue boxes denote coding exonic sequence, light blue boxes are non-coding exonic sequence and dark blue lines are introns. (B and C) RNA-seq data from CLL cases show no differences in the inclusion of coding exon 2 based on *k*-mer counts from homozygotes for the rs539846 CLL risk allele (AA, N=20) and homozygotes for the non-risk allele (CC, N=10). Data shown are mean percentage k-mer counts for each splice junction ± standard deviation. *P*-values were determined with a Student's *t*-test. No differences in splicing levels were observed for other *BMF* exons and little support was found for the presence of the short coding exon 2 isoform (*k*-mer counts <5 per sample).

Table S1, related to Figure 1. GWAS *P*-values and functional annotation of SNPs in LD (r^2 > 0.2) with rs539846

SNP	Position[a]	*P*-value[b]	LD[c]	Conservation: GERP	Conservation: PhastCons	Functional annotation[d]: DNase	H3K4Me1	H3K4Me3	H3K27Ac	Chromatin state	Proteins bound
rs201098886	40385484	0.7	0.22	-9.12	0.00					4-Strong enhancer	
rs11637595	40387728	2.64E-05	0.35	-2.36	0.00					4-Strong enhancer	
rs11637681	40387971	2.21E-05	0.35	-0.31	0.00					4-Strong enhancer	
rs11634257	40388492	1.44E-05	0.35	4.54	0.86					4-Strong enhancer	POL2, POL24H8
rs35603048	40391965	2.83E-05	0.32	0.65	0.00					4-Strong enhancer	
rs11631335	40395604	1.01E-05	0.22	4.82	0.50					4-Strong enhancer	
rs34231574	40396001	6.53E-13	0.8	-0.15	0.49					4-Strong enhancer	
rs12148376	40397094	0.001	0.33	1.87	0.97					4-Strong enhancer	CTCF, ELF1, PAX5C20, RAD21, SMC3, ZNF143
rs34245505	40397191	4.59E-05	0.21	-0.73	0.00					4-Strong enhancer	CTCF, ELF1, PAX5C20, RAD21, SMC3, ZNF143
rs62018159	40397421	6.59E-06	0.23	4.41	0.99					4-Strong enhancer	
**rs539846**	**40397936**	**1.42E-13**	**1**	**4.81**	**1.00**					**4-Strong enhancer**	**NFKB (RELA), POL2, BCL3, TBP, POL24H8**
rs56248706	40398752	-	0.40	0.67	0.00					4-Strong enhancer	
rs484943	40398754	0.002	0.40	1.44	0.00					4-Strong enhancer	
rs35477346	40400195	2.69E-04	0.41	-0.69	0.00					4-Strong enhancer	
rs36081508	40403019	1.44E-12	0.94	1.02	0.34					11-Weak transcribed	
rs8024033	40403657	7.93E-13	0.91	-0.67	0.01					11-Weak transcribed	
rs62019961	40404898	1.70E-06	0.22	-0.06	0.16					7-Weak enhancer	
rs580781	40405150	9.01E-07	0.31	2.00	0.00					6-Weak enhancer	BCL3
rs1471584	40407191	2.09E-06	0.29	0.83	0.01					7-Weak enhancer	
rs537455	40407824	3.66E-06	0.30	1.57	0.85					4-Strong enhancer	

				Conservation		Functional annotation[d]					
SNP	Position[a]	*P*-value[b]	LD[c]	GERP	PhastCons	DNase	H3K4Me1	H3K4Me3	H3K27Ac	Chromatin state	Proteins bound
rs77590215	40409331	1.16E-06	0.22	3.23	0.00					11-Weak transcribed	
rs8038982	40409985	1.14E-06	0.20	-2.04	0.00					11-Weak transcribed	
rs8033229	40411884	1.26E-06	0.30	0.74	0.03					13-Heterochromatin	
rs149613511	40414024	1.22E-06	0.22	3.30	0.98					13-Heterochromatin	
rs8023845	40414116	9.23E-07	0.22	-1.86	0.00					13-Heterochromatin	
rs9635304	40415932	1.19E-06	0.30	-0.09	0.01					13-Heterochromatin	
rs8031450	40418598	1.66E-06	0.31	0.05	0.01					13-Heterochromatin	
rs534304	40420433	2.66E-06	0.31	0.00	0.00					13-Heterochromatin	
rs673328	40420928	2.52E-06	0.31	0.00	0.00					13-Heterochromatin	
rs548686	40421006	2.78E-04	0.24	0.00	0.00					13-Heterochromatin	
rs4924423	40437924	0.06	0.23	-3.17	0.00					13-Heterochromatin	

LD, linkage disequilibrium; GERP, Genomic Evolutionary Rate Profiling; data for rs539846 are emboldened

[a] Chromosome 15 genomic position (human genome NCBI build 37)

[b] *P*-values from meta-analysis of imputed GWAS UK-CLL1 and UK-CLL2

[c] LD value (r^2) between listed SNP and rs539846, derived from 1000 Genomes European populations

[d] Epigenetic annotations are derived from ENCODE project data on GM12878. DNaseI hypersensitivity (grey) and histone modification (yellow enhancer marks, red promoter marks) peaks are indicated by shaded boxes. Annotated chromatin states were determined by the ChromHMM 15-state model (Ernst and Kellis, 2012).

Table S2, related to Figure 4. Expression analysis based on Affymetrix U219 array data from primary CLL cases. (A) Spearman's rho correlation between BMF (11737439_x_at) and RELA (11718103_a_at) expression; (B) eQTL analysis for rs539846 and genes within 1Mb with P ≤ 0.05

(A)

	BMF (11737439_x_at) / RELA (11718103_a_at)
Correlation coefficient	0.14
P-value (two-tailed)	0.004
N	429

Affymetrix probe set names in brackets

(B)

SNP tested	Probe	Gene	t-statistic	*P*-value	FDR	Beta
rs539846	11737439_x_at	*BMF*	-3.61	0.0003	0.02	-0.06
rs539846	11737440_a_at	*BMF*	-3.32	0.0010	0.04	-0.21
rs539846	11720647_a_at	*BUB1B*	-2.66	0.0081	0.19	-0.09
rs539846	11750060_a_at	*BMF*	-2.56	0.0107	0.19	-0.05
rs539846	11759328_at	*CASC5*	2.08	0.0385	0.50	0.16
rs539846	11739650_at	*DLL4*	-2.05	0.0413	0.50	-0.03

Table S3, related to Figure 4. Relationship between rs539846 and CLL prognostic factors in UK-GWAS and ICGC datasets

	Variable		Genotype			
			CC	CA	AA	*P*-value
	IGHV status	Mutated	56	111	92	0.15
		Unmutated	31	123	86	
	Trisomy 12	No trisomy 12	27	80	72	0.17
		Trisomy 12	4	15	5	
	13q14 deletion	Undeleted	9	44	32	0.44
		Deleted	22	51	45	
	6q21 deletion	Undeleted	24	71	47	0.17
		Deleted	1	6	7	
	11q23 deletion	Undeleted	28	76	61	0.26
		Deleted	3	19	16	
UK-GWAS	17p13 deletion	Undeleted	30	85	70	0.72
		Deleted	1	8	5	
	CD38 expression	Low	10	27	20	0.26
		High	14	57	50	
	ZAP70 expression	Low	15	41	32	0.43
		High	14	57	50	
	CLLU1 expression	Low	13	39	38	0.25
		High	18	48	34	
	NOTCH1 mutation	Unmutated	21	69	61	0.61
		Mutated	2	10	5	
	SF3B1 mutation	Unmutated	16	66	49	0.93
		Mutated	4	11	10	
ICGC	*IGHV* status	Mutated	38	149	100	0.74
		Unmutated	20	82	64	

Table S4, related to Figure 4. Relationship between (A) rs539846 and patient outcome and (B) *BMF* transcript levels and patient outcome in UK-GWAS and ICGC datasets

(A)

	Dataset	Genotype			*P*-value
		CC	CA	AA	
Deaths/All cases	UK-GWAS	11/59	31/238	20/166	0.17
	ICGC	28/34	84/102	68/83	0.97

(B)

BMF transcript levels		Deaths/All cases	*P*-value
Quartile[a]	1	13/101	0.45
	2	10/110	
	3	13/105	
	4	16/110	

[a]Quartile 1 to 4 correspond to lowest to highest *BMF* mRNA levels

Table S5, related to Experimental Procedures. Sequences of primers used in experiments

4C primers[a]	
Reading primer	AATGATACGGCGACCACCGAGATCTACACTCTTTCCCTACACGACGCTCTTCCGATCTTAGTCAGACACCCAGATC
Non reading primer	CAAGCAGAAGACGGCATACGAGATCGGTCTCGGCATTCCTGCTGAACCGCTCTTCCGATCTAGAACCCCCAGGAATCTCTA

siRNA oligos	
p65_siRNA	GCAUUAACUUCUCUGGAAAUU
Control siRNA	AGGUAGUGUAAUCGCCUUG

Plasmid construct and sequencing primers	
BMF_F	GGGCAACAGGAGCCACTTTA
BMF_R	TGGAGGAGCTGGAGGATGAT

qPCR primers	
RELA_F	CCCACGAGCTTGTAGGAAAGG
RELA_R	GGATTCCCAGGTTCTGGAAAC
BMF_F	TCTCTGCTGACCTGTTTGCC
BMF_R	TCTGGGTAGCTTTGTCTTCCT
GAPDH_F	GAAGGTGAAGGTCGGAGTC
GAPDH_R	GAAGATGGTGATGGGATTTC

[a]Designed using 4C primer design program (http://mnlab.uchicago.edu/4Cpd/)

SUPPLEMENTAL EXPERIMENTAL PROCEDURES

Germline exome sequencing in CLL families

Patients included in this study were enrolled in a UK national study of CLL genetics established by the Institute of Cancer Research Divisions of Genetics and Epidemiology and Molecular Pathology in 1996. The diagnosis of CLL and other haematological cancers in family members were established. In all cases the diagnosis of CLL was based on accepted standard clinico-pathological and immunological criteria that are in accordance with current World Health Organization classification guidelines. Informed consent was obtained under MREC 99/1/082.

In total, 141 CLL patients from 66 families were subject to exome sequencing on an Illumina HiSeq2000 analyser following exon capture using the Nextera Rapid Capture Exome Enrichment kit (Illumina). Paired-end fastq files were extracted using CASAVA software (version 1.8.1, Illumina) and aligned to build 37 of the human reference genome using Stampy (Lunter and Goodson, 2011) and BWA software (Li and Durbin, 2009). Alignments were processed using the Genome Analysis Tool Kit pipeline (version 3.2-2, (McKenna et al., 2010; Van der Auwera et al., 2013)), according to best practices. Variants were filtered for positions found in >1 sample from an in-house collection of 1,609 control exomes including; 961 samples from the ICR1000 dataset (Ruark et al., 2015) generated by Professor Nazneen Rahman's Team in the Division of Genetics & Epidemiology at The Institute of Cancer Research, London plus an extra 648 samples from the UK 1958 Birth Cohort (Power and Elliott, 2006), sequenced in-house using Illumina TruSeq exome methodology. Positions resulting in protein-altering changes were identified using the Ensembl Variant Effect Predictor (version 78) and variants shared between family members were annotated using custom scripts.

SUPPLEMENTAL REFERENCES

Lunter, G., and Goodson, M. (2011). Stampy: a statistical algorithm for sensitive and fast mapping of Illumina sequence reads. Genome Res *21*, 936-939.

McKenna, A., Hanna, M., Banks, E., Sivachenko, A., Cibulskis, K., Kernytsky, A., Garimella, K., Altshuler, D., Gabriel, S., Daly, M.*, et al.* (2010). The Genome Analysis Toolkit: a MapReduce framework for analyzing next-generation DNA sequencing data. Genome Res *20*, 1297-1303.

Power, C., and Elliott, J. (2006). Cohort profile: 1958 British birth cohort (National Child Development Study). Int J Epidemiol *35*, 34-41.

Ruark, E., Münz, M., Renwick, A., Clarke, M., Ramsay, E., Hanks, S., Mahamdallie, S., Elliott, A., Seal, S., Strydom, A.*, et al.* (2015). The ICR1000 UK exome series: a resource of gene variation in an outbred population. F1000 Research.

Van der Auwera, G.A., Carneiro, M.O., Hartl, C., Poplin, R., Del Angel, G., Levy-Moonshine, A., Jordan, T., Shakir, K., Roazen, D., Thibault, J.*, et al.* (2013). From FastQ data to high confidence variant calls: the Genome Analysis Toolkit best practices pipeline. Curr Protoc Bioinformatics *11*, 11.10.11-11.10.33.
