# Supplementary material for: Genetic Predisposition to Chronic Lymphocytic Leukemia Is Mediated by a BMF Super-Enhancer Polymorphism
Source: Cell Rep. 2016 Aug 11;16(8):2061–7. doi: 10.1016/j.celrep.2016.07.053 (PMC4999417; doi:10.1016/j.celrep.2016.07.053)
Supplement: Document S2. Article plus Supplemental Information [file mmc2.pdf]

Cell Reports

Report

Genetic Predisposition to Chronic Lymphocytic Leukemia Is Mediated by a *BMF* Super-Enhancer Polymorphism

Graphical Abstract

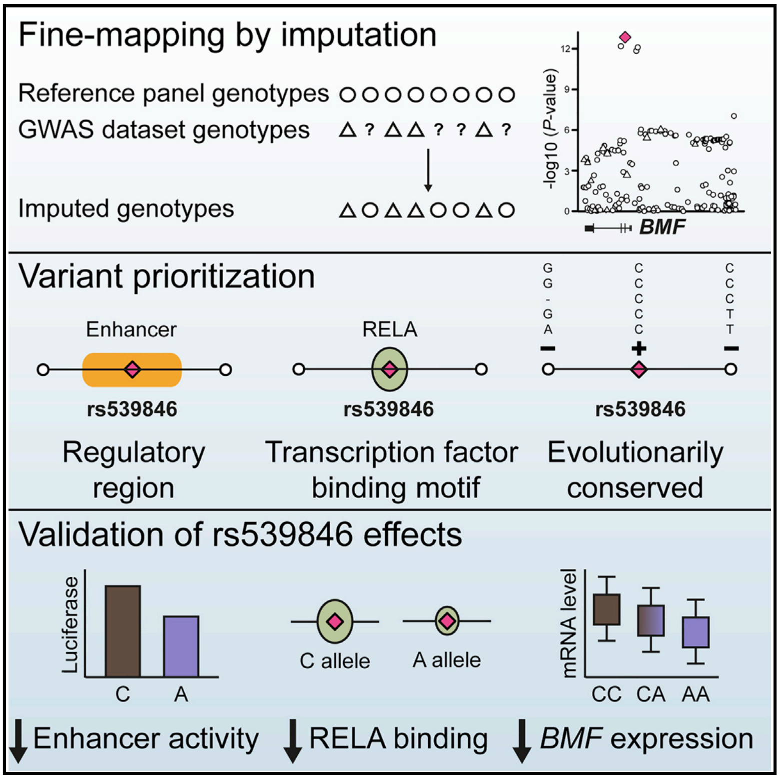

Highlights

- SNP rs539846 underlies 15q15.1 association with chronic lymphocytic leukemia
- rs539846 resides in a B cell super-enhancer, disrupting a conserved RELA-binding site
- The rs539846 risk allele (A) reduces enhancer activity and RELA binding in CLL
- rs539846-A confers lower *BMF* expression

Authors

Radhika Kandaswamy, Georgina P. Sava, Helen E. Speedy, ..., James M. Allan, Elías Campo, Richard S. Houlston

Correspondence

richard.houlston@icr.ac.uk

In Brief

Kandaswamy et al. find that SNP rs539846 underlies the 15q15.1 chronic lymphocytic leukemia risk locus. Follow-up data demonstrate that rs539846 resides within a transcriptional enhancer and alters RELA binding at a conserved site. The rs539846-A risk allele results in reduced RELA-mediated enhancer activity and lower expression of BCL-2-modifying factor.

Kandaswamy et al., 2016, Cell Reports *16*, 2061–2067
August 23, 2016 © 2016 The Author(s).
http://dx.doi.org/10.1016/j.celrep.2016.07.053

Cell Reports
Report

OPEN ACCESS
CellPress

Genetic Predisposition to Chronic Lymphocytic Leukemia Is Mediated by a *BMF* Super-Enhancer Polymorphism

Radhika Kandaswamy,[1,8] Georgina P. Sava,[1,8] Helen E. Speedy,[1,8] Sílvia Beà,[2] José I. Martín-Subero,[2,3] James B. Studd,[1] Gabriele Migliorini,[1] Philip J. Law,[1] Xose S. Puente,[4] David Martín-García,[2] Itziar Salaverria,[2] Jesús Gutiérrez-Abril,[4] Carlos López-Otín,[4] Daniel Catovsky,[5] James M. Allan,[6] Elías Campo,[7] and Richard S. Houlston[1,5,9,*]
[1]Division of Genetics and Epidemiology, The Institute of Cancer Research, London SW7 3RP, UK
[2]Institut d'Investigacions Biomèdiques August Pi i Sunyer (IDIBAPS), 08036 Barcelona, Spain
[3]Departament d'Anatomía Patològica, Microbiología i Farmacología, Universitat de Barcelona, 08036 Barcelona, Spain
[4]Departamento de Bioquímica y Biología Molecular, Instituto Universitario de Oncología (IUOPA), Universidad de Oviedo, 33006 Oviedo, Spain
[5]Division of Molecular Pathology, The Institute of Cancer Research, London SW7 3RP, UK
[6]Newcastle Cancer Centre, Northern Institute for Cancer Research, Medical School, Newcastle University, Newcastle-upon-Tyne NE2 4HH, UK
[7]Unitat de Hematología, Hospital Clínic, IDIBAPS, Universitat de Barcelona, 08036 Barcelona, Spain
[8]Co-first author
[9]Lead Contact
*Correspondence: richard.houlston@icr.ac.uk
http://dx.doi.org/10.1016/j.celrep.2016.07.053

SUMMARY

Chronic lymphocytic leukemia (CLL) is an adult B cell malignancy. Genome-wide association studies show that variation at 15q15.1 influences CLL risk. We deciphered the causal variant at 15q15.1 and the mechanism by which it influences tumorigenesis. We imputed all possible genotypes across the locus and then mapped highly associated SNPs to areas of chromatin accessibility, evolutionary conservation, and transcription factor binding. SNP rs539846 C>A, the most highly associated variant ($p = 1.42 \times 10^{-13}$, odds ratio = 1.35), localizes to a super-enhancer defined by extensive histone H3 lysine 27 acetylation in intron 3 of B cell lymphoma 2 (BCL2)-modifying factor (*BMF*). The rs539846-A risk allele alters a conserved RELA-binding motif, disrupts RELA binding, and is associated with decreased *BMF* expression in CLL. These findings are consistent with rs539846 influencing CLL susceptibility through differential RELA binding, with direct modulation of *BMF* expression impacting on anti-apoptotic BCL2, a hallmark of oncogenic dependency in CLL.

INTRODUCTION

Although genome-wide association studies (GWASs) frequently have identified statistically significant associations within non-coding regions of the genome, the underlying causal variant has been elucidated in only a few instances. GWASs of chronic lymphocytic leukemia (CLL) have identified 31 risk loci, with the signal annotating B cell lymphoma 2 (BCL2)-modifying factor (*BMF*) at 15q15.1 being highly robust (Berndt et al., 2013, 2016; Crowther-Swanepoel et al., 2010; Di Bernardo et al., 2008; Slager et al., 2011, 2012; Speedy et al., 2014).

Elevated expression of the anti-apoptotic protein BCL2 is a hallmark of CLL, driving the accumulation of mature leukemic lymphocytes (Hanada et al., 1993). BMF, a BH3-only pro-apoptotic member of the BCL2 protein family, neutralizes the anti-apoptotic activity of BCL2 through direct interaction (Puthalakath et al., 2001). Here we sought to identify the causal polymorphism(s) driving the 15q15.1 association with CLL susceptibility as a basis for understanding BCL2 addiction mechanisms in CLL.

RESULTS

Fine-Mapping of the 15q15.1 CLL Risk Locus

A previous GWAS reported an association between rs8024033 at 15q15.1 and CLL risk (Berndt et al., 2013). To refine the association signal, we performed fine-mapping of the 15q15.1 CLL risk locus by imputation of our European GWAS to 1000 Genomes Project (Abecasis et al., 2012) and UK10K (UK10K Consortium et al., 2015) reference panels. By this approach, we identified four risk SNPs with minor allele frequency >0.01 and association $p < 5.0 \times 10^{-7}$ (Figure 1A; Table S1). The lead SNP, rs539846 (odds ratio = 1.35, $p = 1.42 \times 10^{-13}$), mapped to the third intron of *BMF* and was in high linkage disequilibrium (LD, $r^2 = 0.91$) with the published SNP, rs8024033. We verified the fidelity of imputed rs539846 genotypes by Sanger sequencing in a subset of 176 CLL cases, demonstrating >95% concordance.

To rule out the existence of multiple statistical signals at the *BMF* locus, we repeated association testing conditional on

This is an open access article under the CC BY license (http://creativecommons.org/licenses/by/4.0/).

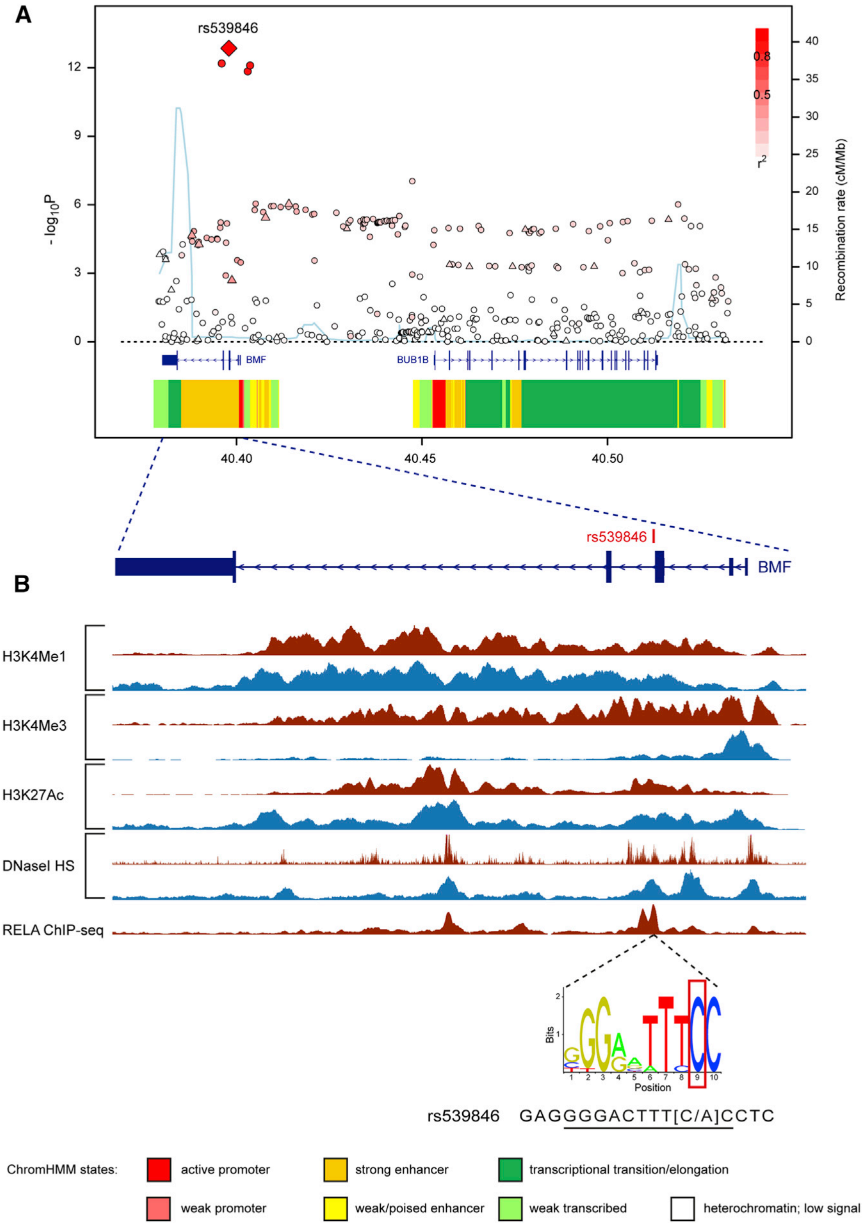

Figure 1. Genetic Mapping and Epigenetic Landscape at the 15q15.1 Locus

(A) SNAP plot of the 15q15.1 chronic lymphocytic leukemia susceptibility locus. Genotyped (triangles) and imputed (dots) SNPs are shown based on their megabase chromosomal position on the x axis and -log10 p value on the y axis. Color intensity of each symbol reflects the extent of LD with rs539846 (white r^2 = 0 to dark red r^2 = to 1). Recombination rates, estimated using HapMap samples of European ancestry, are shown by a light blue line. Physical positions are based on NCBI build 37 of the human genome. Also shown are relative gene positions and chromatin state segmentation (ChromHMM) for GM12878 derived from ENCODE project data.

(B) ChIP-seq data for H3K4Me1, H3K4Me3, and H3K27Ac histone modifications and DNaseI hypersensitivity (HS) are illustrated for GM12878 from ENCODE (brown) and for CLL cells from the Blueprint Project (blue). ChIP-seq data for RELA in GM12878 also is shown. Data are shown relative to the genomic arrangement of *BMF* and were plotted in the University of California, Santa Cruz Genome Browser. Also illustrated are the position-weighted matrix for RELA and the motif sequence (underlined) altered by rs539846 (red box).

rs539846 genotypes, observing no significant variants (most significant variant: rs181168015, p = 1.52 × 10^{-4}; Figure S1). We also found no rare non-synonymous variants in *BMF* in the germline exomes of 141 CLL cases (enriched for genetic susceptibility by virtue of family history; Supplemental Experimental Procedures). Collectively these results are consistent with a single underlying variant at the 15q15.1 locus.

Definition of rs539846 as a Plausible CLL Risk SNP

To further prioritize candidate CLL risk variants, we examined the regulatory potential of SNPs in LD (r^2 > 0.2) with rs539846, based on epigenetic data from lymphoblastoid cell lines (LCLs [ENCODE Project Consortium, 2012]) and primary CLL cells. These data showed that rs539846 resides within an active enhancer region inferred by DNaseI sensitivity and H3K4me1 and H3K27ac histone modifications in both cell types (Figure 1B; Table S1). Moreover, analysis of histone H3K27ac data from lymphoid cells of both B cell and T cell lineages defined a B cell-specific 15q15.1 super-enhancer that spans ~80 kb, encompassing the CLL risk locus (Hnisz et al., 2013), while high-throughput chromosome conformation capture (Hi-C) data from LCLs (Rao et al., 2014) show that this putative super-enhancer element overlaps a chromatin contact domain (Figure 2A).

Since causal SNPs that drive GWAS associations may function by altering transcription factor binding, we examined whether 15q15.1 candidate risk SNPs disrupt predicted JASPAR motifs. This revealed that rs539846 alters a highly conserved base within a putative RELA-binding motif (GGGACTTT[C/A]C, phastCons score = 1.00, Genomic Evolutionary Rate Profiling [GERP] score = 4.81) (Figure 1B). Encyclopedia of DNA Elements (ENCODE) transcription factor chromatin immunoprecipitation sequencing (ChIP-seq) in LCLs confirmed the presence of RELA binding across this site (Figure 1B) in cells homozygous for the rs539846-C allele (non-risk allele, preserving the RELA motif). Within the 15q15.1 chromatin contact domain, chromosome conformation capture–on-chip with sequencing (4C-seq) in the MEC1 CLL cell line showed a high frequency of three-dimensional contacts between the viewpoint (adjacent to

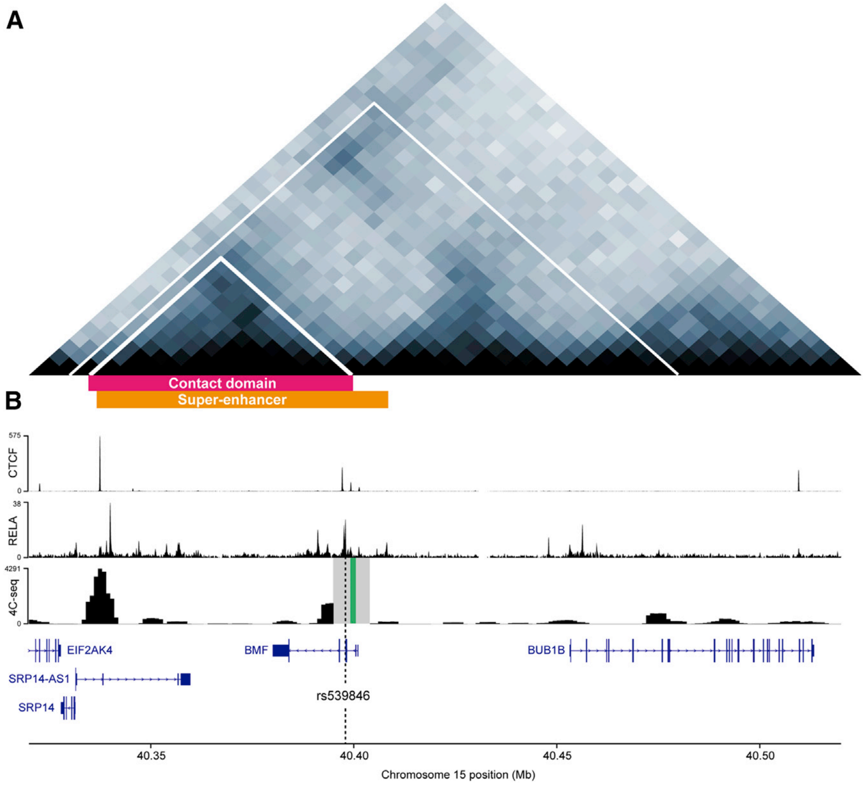

Figure 2. Contact Profile of the *BMF* 4C Viewpoint in Combination with ChIP-Seq Data

(A) Heatmap representing chromatin interactions in GM12878 cells at 40.32–40.52 Mb on chromosome 15. Chromatin contact domains called by the Arrowhead algorithm are marked by white lines (Rao et al., 2014). The contact domain and CD19$^+$ B cell super-enhancer (Hnisz et al., 2013) encompassing rs539846 are labeled in pink and yellow, respectively.

(B) 4C-seq analyses in MEC1 CLL cells indicate the formation of a loop domain between rs539846 and *cis*-regulatory elements. The 4C viewpoint (green box) lies adjacent to rs539846 (dotted line). A 10-kb masked region (gray box) is also marked. ENCODE ChIP-seq data from GM12878 cells show the correspondence between loop formation and CTCF and RELA transcription factor occupancy. Canonical transcripts and chromosome 15 position also are shown.

rs539846) and the distal end of the predicted super-enhancer, with both points overlapping regions of RELA and CTCF binding (Figure 2B).

No other candidate CLL risk variant at the 15q15.1 locus showed the unique combination of evolutionary conservation, active enhancer localization, and disruption of a transcription factor-binding motif, thus re-affirming that rs539846 is the single best causal SNP candidate.

rs539846 Alters RELA-Mediated Enhancer Activity

We next performed luciferase reporter assays to determine the effect of rs539846 on enhancer activity. MEC1 cells transfected with constructs containing the risk A allele demonstrated a significant reduction in normalized luminescence compared to the C allele ($p = 0.015$, Figures 3A and 3B), indicating that the intact RELA motif is required for enhancer activity. We assayed protein-DNA interactions for rs539846-C and -A alleles using electrophoretic mobility shift assays (EMSAs). The C allele formed stronger protein-DNA complexes compared with the A allele (Figure 3C), and in an EMSA super-shift assay RELA was preferentially recruited to the C allele (Figure 3C).

rs539846 Alters RELA-Mediated Regulation of *BMF*

To determine whether *BMF* is a target of RELA-mediated regulation, we first queried the International Cancer Genome Consortium (ICGC) dataset, revealing a correlation between *RELA* and *BMF* expression in CLL (Table S2; $p = 0.004$). To establish a direct relationship between *RELA* and *BMF* expression, we performed small interfering RNA (siRNA) experiments in MEC1 cells, where knockdown of *RELA* was accompanied by a significant reduction in *BMF* mRNA (Figure 4A, $p = 0.02$; Figure S2). We also investigated whether the rs539846 genotype was associated with *BMF* transcript levels in 426 primary CLL cases. We observed a significant dose relationship between the rs539846-A risk allele and reduced *BMF* mRNA ($p = 0.0003$; Figure 4B). No association was seen between the rs539846 genotype and levels of other genes within 1 Mb of the SNP (false discovery rate < 0.05, Table S2). To investigate whether the rs539846 genotype might influence splicing of *BMF*, we examined RNA sequencing (RNA-seq) data from 30 CLL cases, finding no evidence of aberrant splicing. We also found no differences in the splicing levels of known *BMF* exons between the rs539846 risk allele and non-risk allele homozygotes (Figure S3).

Impact of rs539846 on Prognosis and Survival in CLL Patients

CLL can be classified on the basis of several prognostic factors, including immunoglobulin heavy-chain variable (*IGHV*) mutation status; expression levels of CD38, ZAP70, and CLLU1; as well as somatic genomic abnormalities (trisomy 12, 13q14 deletion, 6q21 deletion, 11q23 deletion, 17p13 deletion, *NOTCH1* mutation, and *SF3B1* mutation). We found no association between the rs539846 genotype and these features in a subset of UK-GWAS and ICGC study cases (Table S3). There was also no association between rs539846 and overall patient outcome (Table S4), and we noted that *BMF* transcript levels were not associated with patient survival (Table S4).

DISCUSSION

Collectively, our data demonstrate that the underlying molecular mechanism for the 15q15.1 CLL risk locus is mediated through

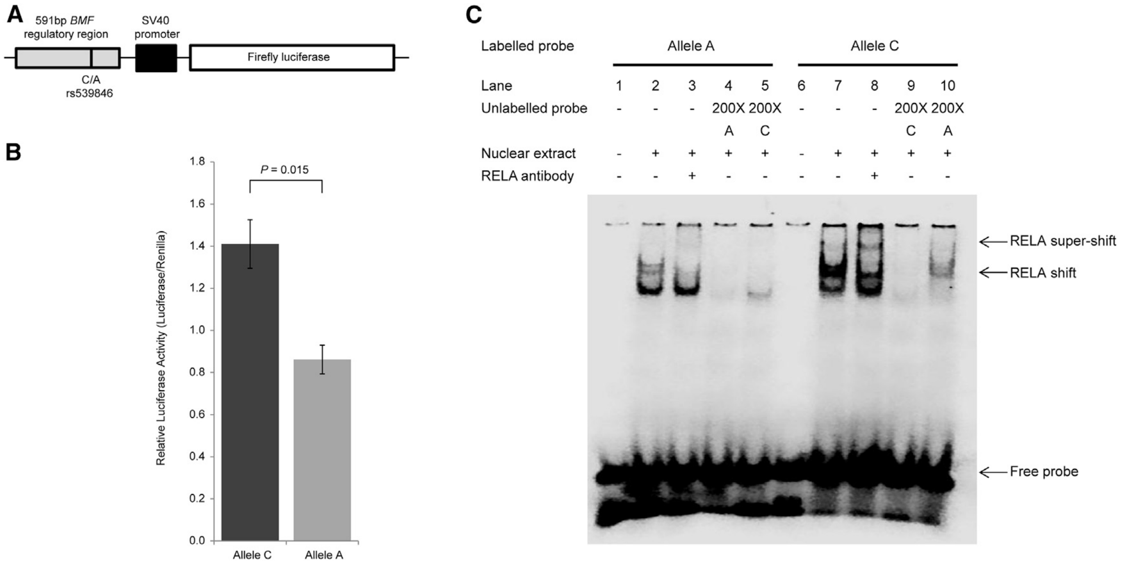

Figure 3. rs539846 Affects RELA-Bound Enhancer Activity

(A) Allele-specific constructs containing a 591-bp putative regulatory sequence flanking rs539846 were cloned into the pGL3-promoter luciferase reporter vector.

(B) The ratio of luminescence from the experimental pGL3-rs539846 constructs to the Renilla internal control, pRL-SV40, was normalized to the empty pGL3-SV40 vector. Data shown are mean ± SE from three independent experiments performed in triplicate. Difference in expression was assessed by the Student's t test. The rs539846-A risk allele had significantly decreased enhancer activity over the protective allele.

(C) EMSA showing differential binding of MEC1 nuclear protein to the rs539846-C allele (protective) and the rs539846-A allele (risk). Binding of double-stranded A allele and C allele probes to MEC1 nuclear extract shows a marked reduction of DNA-protein binding associated with the A allele.

rs539846, which resides within a transcriptional enhancer and disrupts a conserved RELA transcription factor-binding site. Our data are compatible with the rs539846-A allele conferring increased CLL risk through reduced RELA-mediated expression of the pro-apoptotic BCL2 family gene, *BMF*. Furthermore, epigenetic and chromosome conformation capture data are consistent with rs539846 localizing within a chromatin contact domain, overlapping a B cell super-enhancer (Hnisz et al., 2013). This interval, anchored by divergent CTCF-binding sites, forms a loop domain (Rao et al., 2014), which is expected to bring two regions of RELA binding, separated by a linear distance of around 65 kb, into physical contact close to the promoter of *BMF*.

RELA (also known as p65) is a sub-unit of the necrosis factor kappa B (NF-κB) protein complex. This transcription factor complex regulates expression of genes involved in biological processes, such as proliferation, survival, and inflammation. NF-κB signaling is constitutively active in CLL (Furman et al., 2000), while high levels of the pro-survival gene *BCL2*, an established NF-κB target, are a hallmark of the disease (Scarfò and Ghia, 2013).

Here, we provide direct evidence that *BMF* is transcriptionally regulated by RELA, in keeping with the somewhat counter-intuitive observation that levels of pro-apoptotic BMF are high in CLL (Mackus et al., 2005). In the normal response to cellular stress, BMF interacts with BCL2 at the mitochondrial surface and neutralizes its anti-apoptotic properties (Puthalakath et al., 2001). In CLL, it is hypothesized that, although cells maintain some ability to induce pro-apoptotic BH3-only proteins like BMF in response to oncogenic stress, apoptosis ultimately fails due to overexpression of pro-survival proteins.

Our data suggest that, in individuals carrying the rs539846 risk allele, *BMF* transcript levels are reduced and thus the apoptotic response may be attenuated further. Indeed, previous studies in the myeloma cell line U266 have reported that siRNA-mediated knockdown of *BMF* is associated with a decrease in apoptosis following treatment with arsenic trioxide (Morales et al., 2008), whereas mice lacking *Bmf* develop a B cell lymphadenopathy caused by a resistance of B cells to apoptosis (Labi et al., 2008).

We did not observe an association between the SNP and prognostic markers or patient survival in CLL. This is consistent with differential expression of *BMF* being important in the early phases of CLL rather than disease progression per se. We do, however, acknowledge that our analysis had <50% power to demonstrate a 10% difference in patient outcome, and to robustly determine the relationship between *BMF* expression and patient outcome requires much larger patient cohorts.

Finally, this study underlines the importance of BH3-only proteins such as BMF in CLL development. Recently, a number of BH3 mimetics have been developed as potential therapies for lymphoid malignancies (Billard, 2013). These molecules are designed to mimic endogenous BH3-only proteins and bind to pro-survival members of the BCL2 family, facilitating the induction of apoptosis. One example, ABT-199 (Venetoclax), selectively targets BCL2, and, in recent clinical trials involving relapsed or refractory CLL, patients gave an overall response rate of 79% (Roberts et al., 2016). Our findings thus further demonstrate the utility of association studies to define clinically relevant oncogenic pathways.

OPEN ACCESS
CellPress

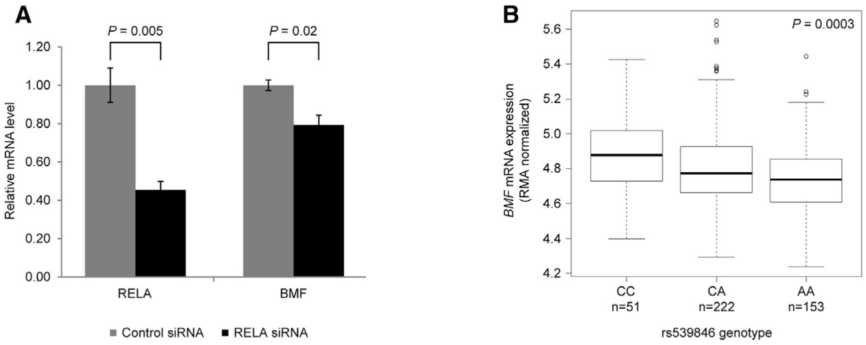

Figure 4. RELA Expression and the rs539846 Genotype Are Correlated with *BMF* Transcript Levels

(A) siRNA knockdown of RELA reduces transcript levels of *BMF*. Data shown are mean ± SE for three independent replicates relative to GAPDH reference mRNA, normalized to control siRNA. The p values were determined with a two-tailed t test.
(B) The rs539846 CLL risk allele (A) was associated with lower *BMF* mRNA levels in 462 patient samples. Boxplot indicates the median (horizontal line), first to third quartiles (box), and 1.5 times the interquartile range (whiskers) of *BMF* expression.

EXPERIMENTAL PROCEDURES

Ethics

Ethical approval for this study was obtained from the UK Multi-Research Ethics Committee (MREC 99/1/082).

Fine-Mapping of the 15q15.1 Locus

We made use of data from two published CLL GWASs: (1) UK-CLL-1 (Di Bernardo et al., 2008), a scan of 517 cases using Illumina HumanCNV370-Duo BeadChips, with Hap1.2M-Duo Custom array data on 2,698 individuals from the Wellcome Trust Case Control Consortium 2 (WTCCC2) 1958 Birth cohort serving as controls; and (2) UK-CLL-2 (Speedy et al., 2014), a scan of 1,403 cases using the Illumina Omni Express BeadChips, with Hap1.2M-Duo Custom array data on 2,501 individuals from the UK Blood Service Control Group serving as controls. Individuals with low call rate (<90%), extremely high or low heterozygosity ($p < 1.0 \times 10^{-4}$), and those evaluated to be of non-European ancestry (using HapMap version 2 populations as a reference) were excluded.

GWAS data were imputed using 1000 Genomes Project phase 1 integrated release 3 (Abecasis et al., 2012), and UK10K 2014 release (UK10K Consortium et al., 2015) as a reference in conjunction with IMPUTE2 v2.1.1 software (Howie et al., 2009). Genotypes were aligned to the positive strand in both imputation and genotyping. Poorly imputed SNPs defined by an information measure, Is < 0.80, were excluded. The association between each SNP and CLL risk was assessed by Cochran-Armitage trend test. To look for independent effects, conditional logistic regression analysis was performed. SNP rs539846 was included as a covariate and association statistics for SNPs within the interval chr15:40379030–40532514 were recalculated (region spans all SNPs in LD $r^2 > 0.2$ with rs539846). To validate imputed rs539846 genotypes, we performed Sanger sequencing in 176 CLL GWAS cases. Primers are listed in Table S5.

Epigenetic Annotation

To explore the epigenetic profile of the interval, we examined LCL chromatin state segmentation, DNase sequencing (DNase-seq), histone modification, and transcription factor ChIP-seq data from the ENCODE project (ENCODE Project Consortium, 2012; Ernst and Kellis, 2012). In addition, ChIP-seq (H3K4me3, H3K4me1, and H3K27ac) and DNase-seq data generated using standard protocols within the Blueprint Consortium, from cells of a CLL patient with mutated *IGHV* (>90% tumor cell content), also were examined (Puente et al., 2015). Detailed protocols are available from the Blueprint Consortium (http://www.blueprint-epigenome.eu). We also used HaploReg (Ward and Kellis, 2012) to examine whether rs539846 or proxy SNPs ($r^2 > 0.2$ in 1000 Genomes EUR reference panel) annotate transcription factor-binding sites or enhancer elements. We assessed sequence conservation using GERP (Cooper et al., 2005) and PhastCons (Siepel et al., 2005). We searched for overlap with annotated super-enhancer regions in lymphoid cell types from B cell ($CD19^+$ and $CD20^+$) and T cell ($CD4^+$ naive and memory; $CD8^+$ naive and memory) lineages (Hnisz et al., 2013).

Hi-C and Definition of a Topological Domain at the 15q15.1 Locus

We made use of publicly available Hi-C data on GM12878 cells (Rao et al., 2014), based on combined replicates digested using MboI, analyzed using the balanced Knight-Ruiz normalization method (Knight and Ruiz, 2013) with a uniform resolution of 5 kb. Contact domains were defined with the Arrowhead algorithm (Rao et al., 2014).

Cell Culture

MEC1 (human CLL) cells were grown in Iscove's modified Dulbecco's medium (Life Technologies) supplemented with 10% fetal calf serum.

4C-Seq

4C-seq libraries were prepared as described (van de Werken et al., 2012), using ten million MEC1 cells cross-linked with 2% formaldehyde. Using 4C primer design software (http://mnlab.uchicago.edu/4Cpd/), we identified a viewpoint adjacent to SNP rs539846. Primary and secondary restriction enzymes were *Dpn*II and *Hind*III (cut sites, chr15:40,397,659–40,397,662 bp and chr15:40,396,692–40,396,697 bp, respectively). Primers are listed in Table S5. Libraries were sequenced on an Illumina MiSeq to obtain 150-bp single-end reads. Reads were mapped to the human genome using Bowtie (version 2.1.0) and filtered for PHRED score < 30. Implementing standard procedures, unique 4C-seq reads were allocated to blind and non-blind fragments. Profiles for the two classes of fragments were obtained at 100-bp resolution and an average profile for a 5-kb running window was computed. For data visualization, we used Vispig (Scales et al., 2014) and incorporated processed ChIP-seq data from the ENCODE Project (ENCODE Project Consortium, 2012).

Plasmid Construction and Luciferase Assays

Allele-specific fragments of a 591-bp region spanning rs539846 were amplified from human genomic DNA using primers detailed in Table S5, cloned into the PCR8/GW/TOPO vector, and then transferred into pGL3 *luc2* promoter vector using Gateway technology (Life Technologies). Reporter constructs were introduced into MEC1 cells by nucleofection, using program X-01 on the Amaxa Nucleofector I (Amaxa Biosystems). Typically, 5×10^6 cells were resuspended in 100 μl Cell Line Nucleofector Solution V and mixed with 3 μg reporter plasmid DNA and 60 ng internal control plasmid (pRL-SV40). Transiently transfected cells were grown for 24 hr before assaying with the Dual-Luciferase Reporter Assay System (Promega) and the Fluoroskan Ascent FL plate reader (Labsystems). Relative luciferase activity was calculated as the ratio of luminescence from the experimental reporter to that of the control reporter. Each transfection experiment was repeated three times and statistical significance was calculated using the Student's t test.

EMSA

Nuclear protein was extracted from MEC1 cells using NE-PER nuclear and cytoplasmic extraction kits (Thermo Fisher Scientific). Infrared dye DY-682-labeled (Eurofins Genomics) and unlabeled (Life Technologies) complementary oligonucleotides flanking rs539846 (5′-GAGGGGACTTT[C/A]CCTCCCC AAAC-3′ and 5′-GTTTGGGGAGG[G/T]AAAGTCCCCTC-3′) were annealed to generate double-stranded EMSA probes. Each 20 μl binding reaction

contained 50 fmol labeled target DNA, 1× binding buffer (10 mM Tris, 50 mM KCl, 1 mM DTT [pH 7.5], 1 μg poly [dI.dC, Sigma-Aldrich], 2.5 mM DTT, and 10 μg nuclear protein extract). The reaction mix was incubated in the dark for 30 min at room temperature. Competition assays were performed by adding 200-fold molar excess of unlabeled probes to the binding reaction. Supershift EMSAs were conducted by adding 2 μg RELA antibody (Santa Cruz Biotechnology) to the binding reaction and incubating for 15 min prior to the addition of labeled probe. Post-incubation, 2 μl 10× Orange loading dye (LI-COR Biosciences) was added to the reaction mix, and the DNA-protein complexes were resolved by electrophoresis on a 6% DNA retardation gel (Life Technologies) in 0.5× Tris-borate-EDTA (TBE) at 4°C. Gels were imaged using the Odyssey Fc Infrared Imaging System (LI-COR Biosciences).

Gene Expression and Splicing Analysis

We used Spearman's rank correlation to assess the relationship between *BMF* and *RELA* transcript levels in the ICGC dataset (Puente et al., 2015). Expression quantitative trait locus analyses were performed for all genes in the 1-Mb region around rs539846, using Affymetrix Human Genome Array U219 data on 426 CLL patients (Puente et al., 2015). Four cases with 15q15.1 copy number losses were excluded. Genotypes were determined by imputation as described and were confirmed from whole-genome-sequencing (WGS) data in 145 samples, with >99% concordance. The association between SNP genotype and expression was evaluated by linear regression controlling for false discovery using matrixEQTL (Shabalin, 2012) implemented in R (version 3.2.0). To assess the impact of rs539846 on splicing, we used RNA-seq data from CLL tumors (Puente et al., 2015), counting individual k-mers supporting each of the possible splicing events. Sample genotype for SNP rs539846 was determined from WGS data, and differences between k-mer counts for rs539846-CC and -AA homozygotes were evaluated using a Student's t test.

The accession numbers for the data utilized in this paper are European Genome-phenome Archive EGAD00010000875 and EGAS00000000092.

siRNA Knockdown

siRNA targeting RELA and a control siRNA (Table S5) were obtained from Eurofins Genomics. MEC1 cells were transfected with 100 nM siRNA using nucleofection as described. Total RNA was extracted 24 hr post-transfection using the RNeasy Plus Mini Kit (QIAGEN). The cDNA was produced using SuperScript II Reverse Transcriptase (Life Technologies). Knockdown efficiency was measured by qPCR and western blot using standard protocols. RELA antibody was used with GAPDH antibody (FL-335; sc-25778, horseradish peroxidase [HRP]; Santa Cruz Biotechnology) as the loading control. Transcript levels of *RELA* and *BMF* were quantified using SYBR Green PCR mastermix (Life Technologies) and normalized to *GAPDH*. The experiment was repeated three times. Primer sequences are detailed in Table S5.

Association between the rs539846 Genotype and Clinical Variables

Logistic regression was used to test the association between the rs539846 genotype and prognostic factors. Trisomy 12, 13q14 deletion, 6q21 deletion, 11q23 deletion, 17p13 deletion, CD38 expression, ZAP70 expression, CLLU1 expression, *NOTCH1* mutation, and *SF3B1* mutation statuses were determined in a subset of UK-CLL-1 patients who were participants in the LRF CLL4 Trial (Catovsky et al., 2007), as previously described (Gonzalez et al., 2013; Oscier et al., 2010, 2013). *IGHV* mutation status was determined as per BIOMED-2 protocols (van Dongen et al., 2003) in a subset of patients from UK-CLL-1, UK-CLL-2, and the ICGC CLL project (Puente et al., 2015). In accordance with published criteria (van Krieken et al., 2007), we classified sequences with germline homology of ≥98% as unmutated and those with homology <98% as mutated. Survival analysis was performed using data from a subset of UK-CLL-1 and ICGC CLL project cases. Analysis was carried out using the log-rank test (using time from diagnosis to death or censoring at the end of follow-up).

SUPPLEMENTAL INFORMATION

Supplemental Information includes Supplemental Experimental Procedures, three figures, and five tables and can be found with this article online at http://dx.doi.org/10.1016/j.celrep.2016.07.053.

AUTHOR CONTRIBUTIONS

R.S.H. designed the study and drafted the manuscript with contributions from all other authors. R.K., G.P.S., H.E.S., and J.B.S. performed the experiments. P.J.L., H.E.S., R.K., G.P.S., and G.M. performed the bioinformatics analysis. D.C. and J.M.A. performed sample recruitment. R.S.H. obtained financial support. In Spain, S.B., J.I.M.-S., D.M.-G., I.S., X.S.P., and J.G.-A. performed data analysis. C.L.-O. and E.C. supervised the data analysis.

ACKNOWLEDGMENTS

Bloodwise provided principal funding (LRF05001, LRF06002, and LRF13044). We also acknowledge support from Cancer Research UK (C1298/A8362 supported by the Bobby Moore Fund) and the Arbib Charitable Fund. G.P.S. is in receipt of a PhD studentship from The Institute of Cancer Research funded by Cancer Research UK. We also acknowledge support from The Spanish Ministry of Science and Innovation (MICINN) through the Instituto de Salud Carlos III (ISCIII) International Cancer Genome Consortium for Chronic Lymphocytic Leukemia (ICGC-CLL Genome Project). This study uses data generated by the Wellcome Trust Case-Control Consortium 1 and 2. A full list of the investigators who contributed to the generation of data is available at http://www.wtccc.org.uk/. We are grateful to all investigators and patients for their participation. We also thank the clinicians and other staff who contributed to the blood sample and data collection for this study.

Received: April 15, 2016
Revised: June 14, 2016
Accepted: July 20, 2016
Published: August 11, 2016

REFERENCES

Abecasis, G.R., Auton, A., Brooks, L.D., DePristo, M.A., Durbin, R.M., Handsaker, R.E., Kang, H.M., Marth, G.T., and McVean, G.A.; 1000 Genomes Project Consortium (2012). An integrated map of genetic variation from 1,092 human genomes. Nature *491*, 56–65.

Berndt, S.I., Skibola, C.F., Joseph, V., Camp, N.J., Nieters, A., Wang, Z., Cozen, W., Monnereau, A., Wang, S.S., Kelly, R.S., et al. (2013). Genome-wide association study identifies multiple risk loci for chronic lymphocytic leukemia. Nat. Genet. *45*, 868–876.

Berndt, S.I., Camp, N.J., Skibola, C.F., Vijai, J., Wang, Z., Gu, J., Nieters, A., Kelly, R.S., Smedby, K.E., Monnereau, A., et al. (2016). Meta-analysis of genome-wide association studies discovers multiple loci for chronic lymphocytic leukemia. Nat. Commun. *7*, 10933.

Billard, C. (2013). BH3 mimetics: status of the field and new developments. Mol. Cancer Ther. *12*, 1691–1700.

Catovsky, D., Richards, S., Matutes, E., Oscier, D., Dyer, M.J., Bezares, R.F., Pettitt, A.R., Hamblin, T., Milligan, D.W., Child, J.A., et al.; UK National Cancer Research Institute (NCRI) Haematological Oncology Clinical Studies Group; NCRI Chronic Lymphocytic Leukaemia Working Group (2007). Assessment of fludarabine plus cyclophosphamide for patients with chronic lymphocytic leukaemia (the LRF CLL4 Trial): a randomised controlled trial. Lancet *370*, 230–239.

Cooper, G.M., Stone, E.A., Asimenos, G., Green, E.D., Batzoglou, S., and Sidow, A.; NISC Comparative Sequencing Program (2005). Distribution and intensity of constraint in mammalian genomic sequence. Genome Res. *15*, 901–913.

Crowther-Swanepoel, D., Broderick, P., Di Bernardo, M.C., Dobbins, S.E., Torres, M., Mansouri, M., Ruiz-Ponte, C., Enjuanes, A., Rosenquist, R., Carracedo, A., et al. (2010). Common variants at 2q37.3, 8q24.21, 15q21.3 and 16q24.1 influence chronic lymphocytic leukemia risk. Nat. Genet. *42*, 132–136.

Di Bernardo, M.C., Crowther-Swanepoel, D., Broderick, P., Webb, E., Sellick, G., Wild, R., Sullivan, K., Vijayakrishnan, J., Wang, Y., Pittman, A.M., et al. (2008). A genome-wide association study identifies six susceptibility loci for chronic lymphocytic leukemia. Nat. Genet. *40*, 1204–1210.

ENCODE Project Consortium (2012). An integrated encyclopedia of DNA elements in the human genome. Nature *489*, 57–74.

Ernst, J., and Kellis, M. (2012). ChromHMM: automating chromatin-state discovery and characterization. Nat. Methods *9*, 215–216.

Furman, R.R., Asgary, Z., Mascarenhas, J.O., Liou, H.-C., and Schattner, E.J. (2000). Modulation of NF-κ B activity and apoptosis in chronic lymphocytic leukemia B cells. J. Immunol. *164*, 2200–2206.

Gonzalez, D., Else, M., Wren, D., Usai, M., Buhl, A.M., Parker, A., Oscier, D., Morgan, G., and Catovsky, D. (2013). CLLU1 expression has prognostic value in chronic lymphocytic leukemia after first-line therapy in younger patients and in those with mutated IGHV genes. Haematologica *98*, 274–278.

Hanada, M., Delia, D., Aiello, A., Stadtmauer, E., and Reed, J.C. (1993). bcl-2 gene hypomethylation and high-level expression in B-cell chronic lymphocytic leukemia. Blood *82*, 1820–1828.

Hnisz, D., Abraham, B.J., Lee, T.I., Lau, A., Saint-André, V., Sigova, A.A., Hoke, H.A., and Young, R.A. (2013). Super-enhancers in the control of cell identity and disease. Cell *155*, 934–947.

Howie, B.N., Donnelly, P., and Marchini, J. (2009). A flexible and accurate genotype imputation method for the next generation of genome-wide association studies. PLoS Genet. *5*, e1000529.

Knight, P.A., and Ruiz, D. (2013). A fast algorithm for matrix balancing. IMA J. Numer. Anal. *33*, 1029–1047.

Labi, V., Erlacher, M., Kiessling, S., Manzl, C., Frenzel, A., O'Reilly, L., Strasser, A., and Villunger, A. (2008). Loss of the BH3-only protein Bmf impairs B cell homeostasis and accelerates γ irradiation-induced thymic lymphoma development. J. Exp. Med. *205*, 641–655.

Mackus, W.J.M., Kater, A.P., Grummels, A., Evers, L.M., Hooijbrink, B., Kramer, M.H.H., Castro, J.E., Kipps, T.J., van Lier, R.A.W., van Oers, M.H.J., and Eldering, E. (2005). Chronic lymphocytic leukemia cells display p53-dependent drug-induced Puma upregulation. Leukemia *19*, 427–434.

Morales, A.A., Gutman, D., Lee, K.P., and Boise, L.H. (2008). BH3-only proteins Noxa, Bmf, and Bim are necessary for arsenic trioxide-induced cell death in myeloma. Blood *111*, 5152–5162.

Oscier, D., Wade, R., Davis, Z., Morilla, A., Best, G., Richards, S., Else, M., Matutes, E., and Catovsky, D.; Chronic Lymphocytic Leukaemia Working Group, UK National Cancer Research Institute (2010). Prognostic factors identified three risk groups in the LRF CLL4 trial, independent of treatment allocation. Haematologica *95*, 1705–1712.

Oscier, D.G., Rose-Zerilli, M.J., Winkelmann, N., Gonzalez de Castro, D., Gomez, B., Forster, J., Parker, H., Parker, A., Gardiner, A., Collins, A., et al. (2013). The clinical significance of NOTCH1 and SF3B1 mutations in the UK LRF CLL4 trial. Blood *121*, 468–475.

Puente, X.S., Beà, S., Valdés-Mas, R., Villamor, N., Gutiérrez-Abril, J., Martín-Subero, J.I., Munar, M., Rubio-Pérez, C., Jares, P., Aymerich, M., et al. (2015). Non-coding recurrent mutations in chronic lymphocytic leukaemia. Nature *526*, 519–524, advance online publication.

Puthalakath, H., Villunger, A., O'Reilly, L.A., Beaumont, J.G., Coultas, L., Cheney, R.E., Huang, D.C.S., and Strasser, A. (2001). Bmf: a proapoptotic BH3-only protein regulated by interaction with the myosin V actin motor complex, activated by anoikis. Science *293*, 1829–1832.

Rao, S.S., Huntley, M.H., Durand, N.C., Stamenova, E.K., Bochkov, I.D., Robinson, J.T., Sanborn, A.L., Machol, I., Omer, A.D., Lander, E.S., and Aiden, E.L. (2014). A 3D map of the human genome at kilobase resolution reveals principles of chromatin looping. Cell *159*, 1665–1680.

Roberts, A.W., Davids, M.S., Pagel, J.M., Kahl, B.S., Puvvada, S.D., Gerecitano, J.F., Kipps, T.J., Anderson, M.A., Brown, J.R., Gressick, L., et al. (2016). Targeting BCL2 with venetoclax in relapsed chronic lymphocytic leukemia. N. Engl. J. Med. *374*, 311–322.

Scales, M., Jäger, R., Migliorini, G., Houlston, R.S., and Henrion, M.Y. (2014). visPIG–a web tool for producing multi-region, multi-track, multi-scale plots of genetic data. PLoS ONE *9*, e107497.

Scarfò, L., and Ghia, P. (2013). Reprogramming cell death: BCL2 family inhibition in hematological malignancies. Immunol. Lett. *155*, 36–39.

Shabalin, A.A. (2012). Matrix eQTL: ultra fast eQTL analysis via large matrix operations. Bioinformatics *28*, 1353–1358.

Siepel, A., Bejerano, G., Pedersen, J.S., Hinrichs, A.S., Hou, M., Rosenbloom, K., Clawson, H., Spieth, J., Hillier, L.W., Richards, S., et al. (2005). Evolutionarily conserved elements in vertebrate, insect, worm, and yeast genomes. Genome Res. *15*, 1034–1050.

Slager, S.L., Rabe, K.G., Achenbach, S.J., Vachon, C.M., Goldin, L.R., Strom, S.S., Lanasa, M.C., Spector, L.G., Rassenti, L.Z., Leis, J.F., et al. (2011). Genome-wide association study identifies a novel susceptibility locus at 6p21.3 among familial CLL. Blood *117*, 1911–1916.

Slager, S.L., Skibola, C.F., Di Bernardo, M.C., Conde, L., Broderick, P., McDonnell, S.K., Goldin, L.R., Croft, N., Holroyd, A., Harris, S., et al. (2012). Common variation at 6p21.31 (BAK1) influences the risk of chronic lymphocytic leukemia. Blood *120*, 843–846.

Speedy, H.E., Di Bernardo, M.C., Sava, G.P., Dyer, M.J., Holroyd, A., Wang, Y., Sunter, N.J., Mansouri, L., Juliusson, G., Smedby, K.E., et al. (2014). A genome-wide association study identifies multiple susceptibility loci for chronic lymphocytic leukemia. Nat. Genet. *46*, 56–60.

UK10K Consortium, Walter, K., Min, J.L., Huang, J., Crooks, L., Memari, Y., McCarthy, S., Perry, J.R., Xu, C., Futema, M., et al. (2015). The UK10K project identifies rare variants in health and disease. Nature *526*, 82–90.

van de Werken, H.J.G., de Vree, P.J.P., Splinter, E., Holwerda, S.J.B., Klous, P., de Wit, E., and de Laat, W. (2012). 4C technology: protocols and data analysis. In Methods in Enzymology, W. Carl and C.D. Allis, eds. (Academic Press), pp. 89–112.

van Dongen, J.J., Langerak, A.W., Brüggemann, M., Evans, P.A., Hummel, M., Lavender, F.L., Delabesse, E., Davi, F., Schuuring, E., García-Sanz, R., et al. (2003). Design and standardization of PCR primers and protocols for detection of clonal immunoglobulin and T-cell receptor gene recombinations in suspect lymphoproliferations: report of the BIOMED-2 Concerted Action BMH4-CT98-3936. Leukemia *17*, 2257–2317.

van Krieken, J.H., Langerak, A.W., Macintyre, E.A., Kneba, M., Hodges, E., Sanz, R.G., Morgan, G.J., Parreira, A., Molina, T.J., Cabeçadas, J., et al. (2007). Improved reliability of lymphoma diagnostics via PCR-based clonality testing: report of the BIOMED-2 Concerted Action BHM4-CT98-3936. Leukemia *21*, 201–206.

Ward, L.D., and Kellis, M. (2012). HaploReg: a resource for exploring chromatin states, conservation, and regulatory motif alterations within sets of genetically linked variants. Nucleic Acids Res. *40*, D930–D934.

Cell Reports, Volume *16*

Supplemental Information

Genetic Predisposition to Chronic Lymphocytic Leukemia Is Mediated by a *BMF* Super-Enhancer Polymorphism

Radhika Kandaswamy, Georgina P. Sava, Helen E. Speedy, Sílvia Beà, José I. Martín-Subero, James B. Studd, Gabriele Migliorini, Philip J. Law, Xose S. Puente, David Martín-García, Itziar Salaverria, Jesús Gutiérrez-Abril, Carlos López-Otín, Daniel Catovsky, James M. Allan, Elías Campo, and Richard S. Houlston

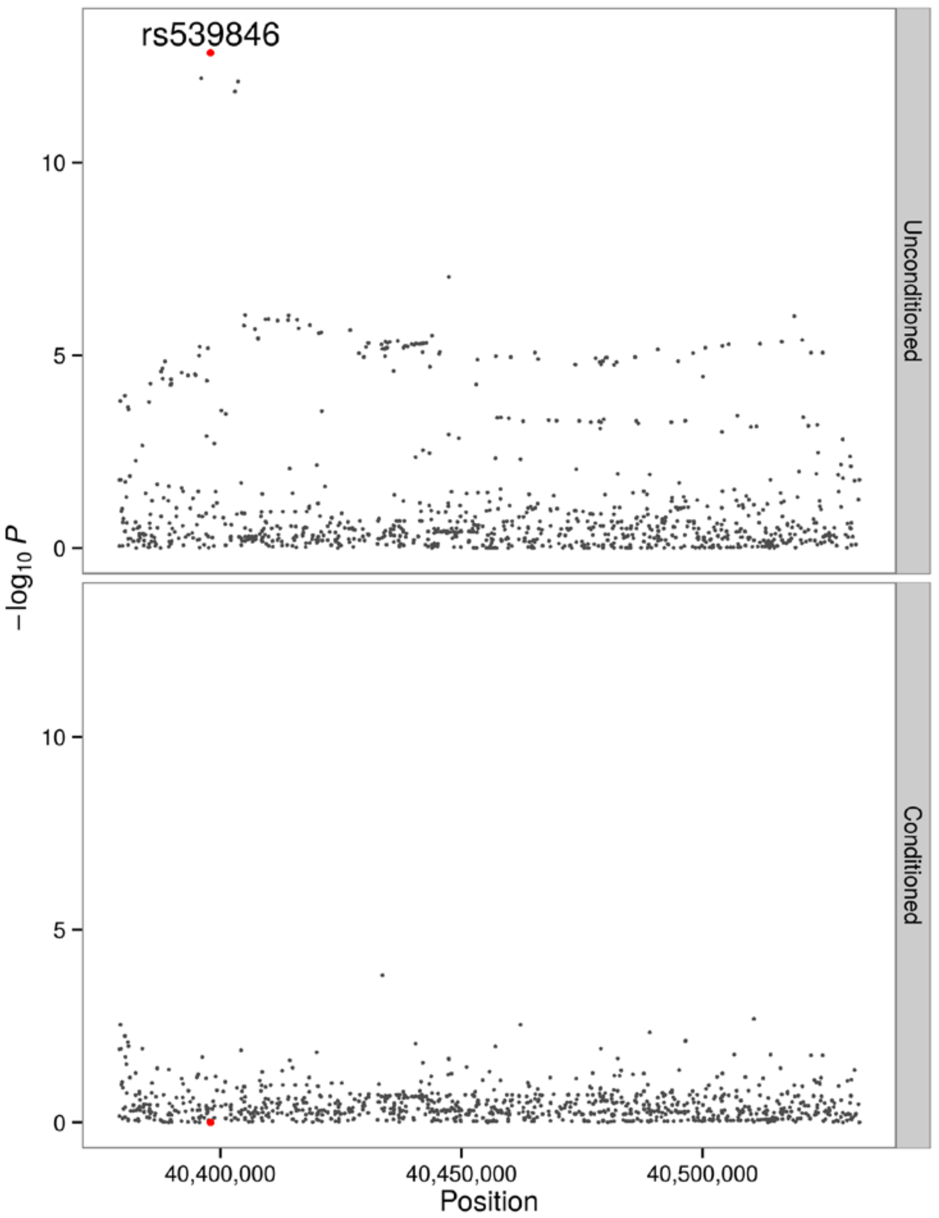

Figure S1, related to Figure 1. Conditional analysis plot for SNP rs539846.

Top panel shows results of unconditioned CLL association analysis for SNPs between 40.38 and 40.53Mb on chromosome 15. Bottom panel shows analysis conditioned on rs539846. SNP position is plotted on the x-axis and –log10 *P*-value is plotted on the y-axis.

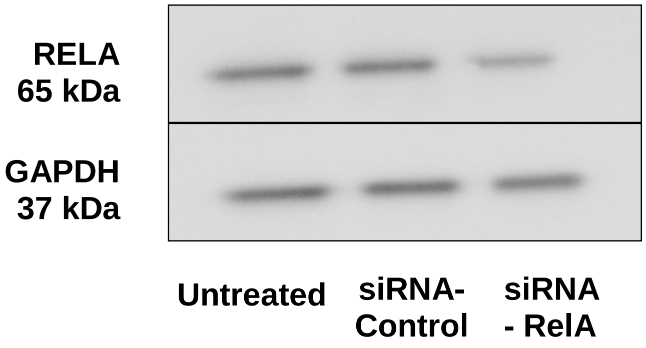

Figure S2, related to Figure 4. siRNA Knockdown of RELA.
Western blot analysis of RELA expression in MEC1 cells 24h post-transfection with indicated siRNAs.

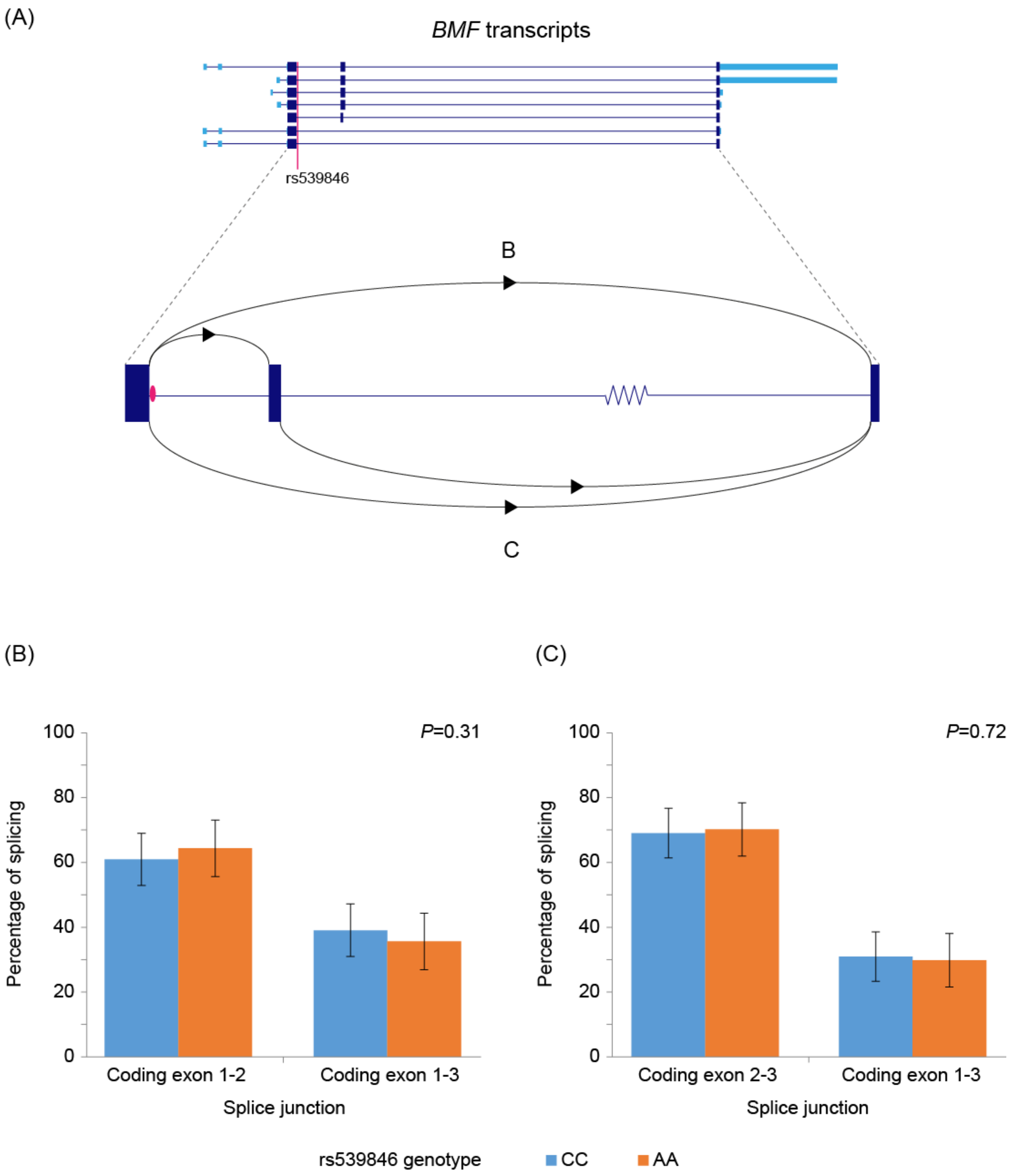

Figure S3, related to Figure 4. *BMF* splicing in CLL cases

(A) Multiple *BMF* transcripts encode three protein isoforms. The second coding exon encodes the BH3 protein domain and may be short-length, full-length or absent. The intronic SNP rs539846 (pink line/dot) resides between coding exon 1 and coding exon 2. Dark blue boxes denote coding exonic sequence, light blue boxes are non-coding exonic sequence and dark blue lines are introns. (B and C) RNA-seq data from CLL cases show no differences in the inclusion of coding exon 2 based on *k*-mer counts from homozygotes for the rs539846 CLL risk allele (AA, N=20) and homozygotes for the non-risk allele (CC, N=10). Data shown are mean percentage k-mer counts for each splice junction ± standard deviation. *P*-values were determined with a Student's *t*-test. No differences in splicing levels were observed for other *BMF* exons and little support was found for the presence of the short coding exon 2 isoform (*k*-mer counts <5 per sample).

Table S1, related to Figure 1. GWAS *P*-values and functional annotation of SNPs in LD (r^2 > 0.2) with rs539846

				Conservation		Functional annotation[d]					
SNP	Position[a]	*P*-value[b]	LD[c]	GERP	PhastCons	DNase	H3K4Me1	H3K4Me3	H3K27Ac	Chromatin state	Proteins bound
rs201098886	40385484	0.7	0.22	-9.12	0.00					4-Strong enhancer	
rs11637595	40387728	2.64E-05	0.35	-2.36	0.00					4-Strong enhancer	
rs11637681	40387971	2.21E-05	0.35	-0.31	0.00					4-Strong enhancer	
rs11634257	40388492	1.44E-05	0.35	4.54	0.86					4-Strong enhancer	POL2, POL24H8
rs35603048	40391965	2.83E-05	0.32	0.65	0.00					4-Strong enhancer	
rs11631335	40395604	1.01E-05	0.22	4.82	0.50					4-Strong enhancer	
rs34231574	40396001	6.53E-13	0.8	-0.15	0.49					4-Strong enhancer	
rs12148376	40397094	0.001	0.33	1.87	0.97					4-Strong enhancer	CTCF, ELF1, PAX5C20, RAD21, SMC3, ZNF143
rs34245505	40397191	4.59E-05	0.21	-0.73	0.00					4-Strong enhancer	CTCF, ELF1, PAX5C20, RAD21, SMC3, ZNF143
rs62018159	40397421	6.59E-06	0.23	4.41	0.99					4-Strong enhancer	
**rs539846**	**40397936**	**1.42E-13**	**1**	**4.81**	**1.00**					**4-Strong enhancer**	**NFKB (RELA), POL2, BCL3, TBP, POL24H8**
rs56248706	40398752	-	0.40	0.67	0.00					4-Strong enhancer	
rs484943	40398754	0.002	0.40	1.44	0.00					4-Strong enhancer	
rs35477346	40400195	2.69E-04	0.41	-0.69	0.00					4-Strong enhancer	
rs36081508	40403019	1.44E-12	0.94	1.02	0.34					11-Weak transcribed	
rs8024033	40403657	7.93E-13	0.91	-0.67	0.01					11-Weak transcribed	
rs62019961	40404898	1.70E-06	0.22	-0.06	0.16					7-Weak enhancer	
rs580781	40405150	9.01E-07	0.31	2.00	0.00					6-Weak enhancer	BCL3
rs1471584	40407191	2.09E-06	0.29	0.83	0.01					7-Weak enhancer	
rs537455	40407824	3.66E-06	0.30	1.57	0.85					4-Strong enhancer	

SNP	Position[a]	*P*-value[b]	LD[c]	Conservation		Functional annotation[d]					
				GERP	PhastCons	DNase	H3K4Me1	H3K4Me3	H3K27Ac	Chromatin state	Proteins bound
rs77590215	40409331	1.16E-06	0.22	3.23	0.00					11-Weak transcribed	
rs8038982	40409985	1.14E-06	0.20	-2.04	0.00					11-Weak transcribed	
rs8033229	40411884	1.26E-06	0.30	0.74	0.03					13-Heterochromatin	
rs149613511	40414024	1.22E-06	0.22	3.30	0.98					13-Heterochromatin	
rs8023845	40414116	9.23E-07	0.22	-1.86	0.00					13-Heterochromatin	
rs9635304	40415932	1.19E-06	0.30	-0.09	0.01					13-Heterochromatin	
rs8031450	40418598	1.66E-06	0.31	0.05	0.01					13-Heterochromatin	
rs534304	40420433	2.66E-06	0.31	0.00	0.00					13-Heterochromatin	
rs673328	40420928	2.52E-06	0.31	0.00	0.00					13-Heterochromatin	
rs548686	40421006	2.78E-04	0.24	0.00	0.00					13-Heterochromatin	
rs4924423	40437924	0.06	0.23	-3.17	0.00					13-Heterochromatin	

LD, linkage disequilibrium; GERP, Genomic Evolutionary Rate Profiling; data for rs539846 are emboldened

[a] Chromosome 15 genomic position (human genome NCBI build 37)

[b] *P*-values from meta-analysis of imputed GWAS UK-CLL1 and UK-CLL2

[c] LD value (r^2) between listed SNP and rs539846, derived from 1000 Genomes European populations

[d] Epigenetic annotations are derived from ENCODE project data on GM12878. DNaseI hypersensitivity (grey) and histone modification (yellow enhancer marks, red promoter marks) peaks are indicated by shaded boxes. Annotated chromatin states were determined by the ChromHMM 15-state model (Ernst and Kellis, 2012).

Table S2, related to Figure 4. Expression analysis based on Affymetrix U219 array data from primary CLL cases. (A) Spearman's rho correlation between BMF (11737439_x_at) and RELA (11718103_a_at) expression; (B) eQTL analysis for rs539846 and genes within 1Mb with P ≤ 0.05

(A)

	BMF (11737439_x_at) / RELA (11718103_a_at)
Correlation coefficient	0.14
P-value (two-tailed)	0.004
N	429

Affymetrix probe set names in brackets

(B)

SNP tested	Probe	Gene	t-statistic	*P*-value	FDR	Beta
rs539846	11737439_x_at	*BMF*	-3.61	0.0003	0.02	-0.06
rs539846	11737440_a_at	*BMF*	-3.32	0.0010	0.04	-0.21
rs539846	11720647_a_at	*BUB1B*	-2.66	0.0081	0.19	-0.09
rs539846	11750060_a_at	*BMF*	-2.56	0.0107	0.19	-0.05
rs539846	11759328_at	*CASC5*	2.08	0.0385	0.50	0.16
rs539846	11739650_at	*DLL4*	-2.05	0.0413	0.50	-0.03

Table S3, related to Figure 4. Relationship between rs539846 and CLL prognostic factors in UK-GWAS and ICGC datasets

	Variable		Genotype			
			CC	CA	AA	*P*-value
UK-GWAS	*IGHV* status	Mutated	56	111	92	0.15
		Unmutated	31	123	86	
	Trisomy 12	No trisomy 12	27	80	72	0.17
		Trisomy 12	4	15	5	
	13q14 deletion	Undeleted	9	44	32	0.44
		Deleted	22	51	45	
	6q21 deletion	Undeleted	24	71	47	0.17
		Deleted	1	6	7	
	11q23 deletion	Undeleted	28	76	61	0.26
		Deleted	3	19	16	
	17p13 deletion	Undeleted	30	85	70	0.72
		Deleted	1	8	5	
	CD38 expression	Low	10	27	20	0.26
		High	14	57	50	
	ZAP70 expression	Low	15	41	32	0.43
		High	14	57	50	
	CLLU1 expression	Low	13	39	38	0.25
		High	18	48	34	
	NOTCH1 mutation	Unmutated	21	69	61	0.61
		Mutated	2	10	5	
	SF3B1 mutation	Unmutated	16	66	49	0.93
		Mutated	4	11	10	
ICGC	*IGHV* status	Mutated	38	149	100	0.74
		Unmutated	20	82	64	

Table S4, related to Figure 4. Relationship between (A) rs539846 and patient outcome and (B) *BMF* transcript levels and patient outcome in UK-GWAS and ICGC datasets

(A)

	Dataset	Genotype			*P*-value
		CC	CA	AA	
Deaths/All cases	UK-GWAS	11/59	31/238	20/166	0.17
	ICGC	28/34	84/102	68/83	0.97

(B)

BMF transcript levels		Deaths/All cases	*P*-value
Quartile[a]	1	13/101	0.45
	2	10/110	
	3	13/105	
	4	16/110	

[a]Quartile 1 to 4 correspond to lowest to highest *BMF* mRNA levels

Table S5, related to Experimental Procedures. Sequences of primers used in experiments

4C primers[a]	
Reading primer	AATGATACGGCGACCACCGAGATCTACACTCTTTCCCTACACGACGCTCTTCCGATCTTAGTCAGACACCCAGATC
Non reading primer	CAAGCAGAAGACGGCATACGAGATCGGTCTCGGCATTCCTGCTGAACCGCTCTTCCGATCTAGAACCCCCAGGAATCTCTA

siRNA oligos	
p65_siRNA	GCAUUAACUUCUCUGGAAAUU
Control siRNA	AGGUAGUGUAAUCGCCUUG

Plasmid construct and sequencing primers	
BMF_F	GGGCAACAGGAGCCACTTTA
BMF_R	TGGAGGAGCTGGAGGATGAT

qPCR primers	
RELA_F	CCCACGAGCTTGTAGGAAAGG
RELA_R	GGATTCCCAGGTTCTGGAAAC
BMF_F	TCTCTGCTGACCTGTTTGCC
BMF_R	TCTGGGTAGCTTTGTCTTCCT
GAPDH_F	GAAGGTGAAGGTCGGAGTC
GAPDH_R	GAAGATGGTGATGGGATTTC

[a]Designed using 4C primer design program (http://mnlab.uchicago.edu/4Cpd/)

SUPPLEMENTAL EXPERIMENTAL PROCEDURES

Germline exome sequencing in CLL families

Patients included in this study were enrolled in a UK national study of CLL genetics established by the Institute of Cancer Research Divisions of Genetics and Epidemiology and Molecular Pathology in 1996. The diagnosis of CLL and other haematological cancers in family members were established. In all cases the diagnosis of CLL was based on accepted standard clinico-pathological and immunological criteria that are in accordance with current World Health Organization classification guidelines. Informed consent was obtained under MREC 99/1/082.

In total, 141 CLL patients from 66 families were subject to exome sequencing on an Illumina HiSeq2000 analyser following exon capture using the Nextera Rapid Capture Exome Enrichment kit (Illumina). Paired-end fastq files were extracted using CASAVA software (version 1.8.1, Illumina) and aligned to build 37 of the human reference genome using Stampy (Lunter and Goodson, 2011) and BWA software (Li and Durbin, 2009). Alignments were processed using the Genome Analysis Tool Kit pipeline (version 3.2-2, (McKenna et al., 2010; Van der Auwera et al., 2013)), according to best practices. Variants were filtered for positions found in >1 sample from an in-house collection of 1,609 control exomes including; 961 samples from the ICR1000 dataset (Ruark et al., 2015) generated by Professor Nazneen Rahman's Team in the Division of Genetics & Epidemiology at The Institute of Cancer Research, London plus an extra 648 samples from the UK 1958 Birth Cohort (Power and Elliott, 2006), sequenced in-house using Illumina TruSeq exome methodology. Positions resulting in protein-altering changes were identified using the Ensembl Variant Effect Predictor (version 78) and variants shared between family members were annotated using custom scripts.

SUPPLEMENTAL REFERENCES

Lunter, G., and Goodson, M. (2011). Stampy: a statistical algorithm for sensitive and fast mapping of Illumina sequence reads. Genome Res *21*, 936-939.

McKenna, A., Hanna, M., Banks, E., Sivachenko, A., Cibulskis, K., Kernytsky, A., Garimella, K., Altshuler, D., Gabriel, S., Daly, M.*, et al.* (2010). The Genome Analysis Toolkit: a MapReduce framework for analyzing next-generation DNA sequencing data. Genome Res *20*, 1297-1303.

Power, C., and Elliott, J. (2006). Cohort profile: 1958 British birth cohort (National Child Development Study). Int J Epidemiol *35*, 34-41.

Ruark, E., Münz, M., Renwick, A., Clarke, M., Ramsay, E., Hanks, S., Mahamdallie, S., Elliott, A., Seal, S., Strydom, A.*, et al.* (2015). The ICR1000 UK exome series: a resource of gene variation in an outbred population. F1000 Research.

Van der Auwera, G.A., Carneiro, M.O., Hartl, C., Poplin, R., Del Angel, G., Levy-Moonshine, A., Jordan, T., Shakir, K., Roazen, D., Thibault, J.*, et al.* (2013). From FastQ data to high confidence variant calls: the Genome Analysis Toolkit best practices pipeline. Curr Protoc Bioinformatics *11*, 11.10.11-11.10.33.
